# Spatio-temporal Model of Endogenous ROS and Raft-Dependent WNT/Beta-Catenin Signaling Driving Cell Fate Commitment in Human Neural Progenitor Cells

**DOI:** 10.1371/journal.pcbi.1004106

**Published:** 2015-03-20

**Authors:** Fiete Haack, Heiko Lemcke, Roland Ewald, Tareck Rharass, Adelinde M. Uhrmacher

**Affiliations:** 1 Modeling and Simulation Group, Institute of Computer Science, University of Rostock, Rostock, Germany; 2 Live Cell Imaging Center, Institute of Biological Sciences, University of Rostock, Rostock, Germany; 3 Reference and Translation Center for Cardiac Stem Cell Therapy (RTC), University Medical Center Rostock, Rostock, Germany; 4 Electrochemical Signaling in Development and Disease, Max-Delbrück-Center for Molecular Medicine (MDC) Berlin-Buch, Berlin-Buch, Germany; University of Virginia, UNITED STATES

## Abstract

Canonical WNT/β-catenin signaling is a central pathway in embryonic development, but it is also connected to a number of cancers and developmental disorders. Here we apply a combined in-vitro and in-silico approach to investigate the spatio-temporal regulation of WNT/β-catenin signaling during the early neural differentiation process of human neural progenitors cells (hNPCs), which form a new prospect for replacement therapies in the context of neurodegenerative diseases. Experimental measurements indicate a second signal mechanism, in addition to canonical WNT signaling, being involved in the regulation of nuclear β-catenin levels during the cell fate commitment phase of neural differentiation. We find that the biphasic activation of β-catenin signaling observed experimentally can only be explained through a model that combines Reactive Oxygen Species (ROS) and raft dependent WNT/β-catenin signaling. Accordingly after initiation of differentiation endogenous ROS activates DVL in a redox-dependent manner leading to a transient activation of down-stream β-catenin signaling, followed by continuous auto/paracrine WNT signaling, which crucially depends on lipid rafts. Our simulation studies further illustrate the elaborate spatio-temporal regulation of DVL, which, depending on its concentration and localization, may either act as direct inducer of the transient ROS/β-catenin signal or as amplifier during continuous auto-/parcrine WNT/β-catenin signaling. In addition we provide the first stochastic computational model of WNT/β-catenin signaling that combines membrane-related and intracellular processes, including lipid rafts/receptor dynamics as well as WNT- and ROS-dependent β-catenin activation. The model’s predictive ability is demonstrated under a wide range of varying conditions for in-vitro and in-silico reference data sets. Our in-silico approach is realized in a multi-level rule-based language, that facilitates the extension and modification of the model. Thus, our results provide both new insights and means to further our understanding of canonical WNT/β-catenin signaling and the role of ROS as intracellular signaling mediator.

## Author Summary

Human neural progenitor cells offer the promising perspective of using in-vitro grown neural cell populations for replacement therapies in the context of neurodegenerative diseases, such as Parkinson’s or Huntington’s disease. However, to control hNPC differentiation within the scope of stem cell engineering, a thorough understanding of cell fate determination and its endogenous regulation is required. Here we investigate the spatio-temporal regulation of WNT/*β*-catenin signaling in the process of cell fate commitment in hNPCs, which has been reported to play a crucial role for the differentiation process of hNPCs. Based on a combined in-vitro and in-silico approach we demonstrate an elaborate interplay between endogenous ROS and lipid raft dependent WNT/beta-catenin signaling controlling the nuclear beta-catenin levels throughout the initial phase of neural differentiation. The stochastic multi-level computational model we derive from our experimental measurements adds to the family of existing WNT models, addressing major biochemical and spatial aspects of WNT/beta-catenin signaling that have not been considered in existing models so far. Cross validation studies manifest its predictive capability for other cells and cell lines rendering the model a suitable basis for further studies also in the context of embryonic development, developmental disorders and cancers.

## Introduction

Canonical WNT signaling is a central pathway in embryonic development and adult homeostasis, while its aberrant form is involved in a number of human cancers and developmental disorders [1–3]. The WNT/*β*-catenin signal transduction is characterized by a reaction cascade, that is initiated by extracellular WNT molecules and eventually leads to an accumulation of cytosolic *β*-catenin and its subsequent shuttling into the nucleus. In the nucleus *β*-catenin associates with the Lef/Tcf transcription factors triggering a pathway-specific gene response relevant for the regulation of various physiological and developmental processes, including neuronal differentiation [3, 4] Accordingly WNT/*β*-catenin signaling has been reported to be involved in the neuronal differentiation process of human neural progenitors cells (hNPCs) [5]. NPCs provide a new, promising basis for the in-vitro growth of neuron populations that can be used in replacement therapies for neurodegenerative diseases, such as Parkinson’s or Huntington’s diseases [6, 7]. However, controlling NPC differentiation in stem cell engineering demands a thorough understanding of neuronal and glial cell fate determination and its endogenous regulation. A first characterization of ReNcell VM197 hNPC cell fate commitment uncovered a spatio-temporal regulation of WNT/*β*-catenin key proteins, like LRP6, DVL, AXIN and *β*-catenin throughout the entire phase of early differentiation [8]. However, the exact mechanisms that drive the WNT/*β*-catenin signaling and therewith control the cell fate commitment in hNPC remain unclear.

One of the key mechanisms of the WNT signal transduction is the formation of a large protein-receptor complex, called signalosome, in response to the extracellular WNT stimulus [9, 10]. The signalosome consists of the membrane-integral receptors FZ and LRP6 and several cytosolic proteins, like Dishevelled (DVL), CK1*γ*, AXIN and GSK-3*β*. The stable aggregation of the signalosome triggers the phosphorylation of several intracellular phosphorylation sites (mainly PPSPXS motifs) in the cytosolic tail of LRP6, generating high-density platforms for the recruitment of AXIN [11–13]. Due to the binding of AXIN (and GSK-3*β*) to LRP6, key components of the destruction complex are inhibited, which in turn leads to an accumulation and translocation of *β*-catenin into the nucleus and eventually to the well known gene transcription signal.

Recently, several studies suggested an involvement of lipid rafts in the WNT/*β*-catenin pathway [14–17]. Lipid rafts are local assemblies of highly concentrated sphingolipids and cholesterol in the cell membrane [18]. The diffusion inside rafts is significantly slowed down, which in turn influences the general diffusion and localization of transmembrane receptors [19, 20]. Apparently, the localization of LRP6 in lipid rafts is crucial for its successful phosphorylation, implying a major impact of lipid rafts on the activation of signalosome, hence WNT/*β*-catenin signaling [15, 17].

Therefore we investigate the mutual influence of lipid rafts on WNT-signaling during the in-vitro differentiation of immortalized human neural progenitor cells (ReNcell VM197). The ReNcell VM197 cell line was derived from the ventral mesencephalon region of a human fetal brain tissue and is characterized by a rapid differentiation. Upon growth factor removal ReNcell VM197 cells differentiate into neurons and glial cells within a few days and without any additional external stimulation. This allows us to study WNT signaling in the context of cell fate commitment in a time dependent manner.

During our investigations we found that lipid raft disruption by Methyl-*β*-Cyclodextrin (MbCD) effectively inhibits WNT/*β*-catenin signal transduction. This implies that raft disruption serves as effective inhibitor for WNT/*β*-catenin signaling in our cell line. However, surprisingly we found that immediately after the initiation of differentiation, raft-deficient cells still show a transient *β*-catenin signaling activity, raising the question what triggers the early immediate response despite the apparent WNT/*β*-catenin signaling inhibition?

In a recent study we showed that during the initiation phase of ReNcell VM197 differentiation an early spontaneous production of reactive oxygen species (ROS) occurs, which promotes a DVL-mediated downstream activation of canonical WNT signaling [21]. ROS are chemically reactive radical and non-radical molecules containing molecular oxygen mainly generated as by-products of the electron transfer pathway in the mitochondrial respiratory chain [22]. Excessive ROS accumulation induces cell damage through an oxidative stress involved in various pathologies as diabetes, cardiovascular diseases or neurological disorders. However, if present in moderate amounts, ROS have been implicated as signaling mediators in various physiological processes i.e. activation of Rac1, PI3K, MAPK cascade, ASK1-dependent apoptosis, p21-mediated signaling, or modulation of thioredoxin-dependent transcription factors [23, 24]. A few recent studies found an involvement of ROS in the regulation of canonical WNT signaling while direct proof that ROS metabolism acts as endogenous transmitters were missing since ROS implication has been reported through the use of exogenous stimulation by pro-oxidant compounds [25] or injury [26]. However further evidence was provided in our previous study on ReNcell VM197 cells, as an early increase of mitochondrial ROS metabolism after growth factor removal was found to modulate DVL-mediated WNT/*β*-catenin pathway and neurogenesis [21].

To evaluate, whether an interplay between ROS-induced and lipid raft dependent WNT/*β*-catenin signaling can explain our experimental results we apply computational modeling. We extend the current standard model of the WNT/*β*-catenin pathway [27] with the aforementioned membrane-related processes including lipid rafts/receptor dynamics and combine this with an intracellular ROS/*β*-catenin signaling mechanism. The model is based on experimental data as well as literature values and has been extensively validated against in-vitro and in-silico data under a wide range of varying conditions.

## Results/Discussion

Computational modeling is increasingly applied to derive or test hypotheses, that in most cases arise from experimental data. Also, simulation experiments are regarded as a valuable complement to wet-lab experiments. The majority of existing WNT models are derived from the Lee model [27, 28]. For a comprehensive overview of existing WNT models the interested reader is referred to a recent review by *Lloyd-Lewis et. al.* [28]. However, most of these models focus on the main intracellular compounds, like *β*-catenin, the destruction complex (typically a simplified version of it), DVL, GSK3*β* and an abstract form of WNT molecules. This also means that most, if not all processes at the membrane are omitted, even though a number of studies demonstrated the crucial role of membrane-related processes in canonical WNT signaling, like receptor activation, aggregation and recruitment of cytosolic proteins like DVL and AXIN [10, 11, 13, 15]. To our knowledge, there exists only one model comprising membrane-related dynamics of WNT signaling [29]. This model neglects important processes like lipid rafts dynamics, receptor clustering and phosphorylation and further employs some unphysiological parameter values, in particular the total number of Frizzled receptors has been fitted to an exceedingly low molecule number, i.e., 30.

To explore the potential mechanisms that drive the spatio-temporal regulation of *β*-catenin signaling during cell fate commitment and to close this important gap in existing models, we built a comprehensive, stochastic WNT/*β*-catenin signaling model, that combines both, membrane-related and intracellular processes. We use literature values as often as possible and fit the remaining parameters to experimental measurements of nuclear *β*-catenin dynamics during in-vitro differentiation of ReNcell VM 197 cells. To further test the calibrated/fitted model we apply cross-validation by reproducing existing in-silico and in-vitro data (measurements of *β*-catenin concentration under different WNT stimuli). However, we also have to verify whether the model predictions are still in accordance with experimental data when it comes to perturbations, like raft disruption. Therefore we analyze the impact of lipid rafts disruption on WNT/*β*-catenin signaling in untreated as well as raft-deficient human progenitor cells during early differentiation.

### Nuclear *β*-catenin dynamics during early differentiation in human neural progenitor cells

In the following we describe experimental data, retrieved from ReNcell VM197 human progenitor cells. The ReNcell VM197 is a well-characterized cell line, that has been successfully applied in several studies and proven to be a simple and accepted model to investigate different aspects of neural differentiation [5, 8, 30–32]. The major advantage of this cell line is its rapid differentiation. Within three days after growth factor removal, ReNcell VM197 cells differentiate into neurons, astrocytes, and oligodendrocytes without any additional exogenous stimulation. We evaluate the impact of lipid raft disruption on WNT/*β*-catenin signaling during differentiation by measuring the temporal progress of WNT signaling in terms of nuclear *β*-catenin concentrations in methyl-*β*-cyclodextrin-treated and untreated cells in the process of cell fate commitment. Accordingly proliferating ReNcell VM197 cells were used as reference (0h), whereas all following time points were measured after initiating the differentiation by growth factor removal. Note, that we only consider the first 12 hours after induction of differentiation. Typically most of the cells commit themselves for differentiation within the first 12 hours. Also, at later time points the cell population of ReNcell VM197 is already so heterogeneous due to differentiation, that potential signal activities may originate from multiple sources.

#### Lipid Rafts Disruption

Before evaluating the potential impact of Lipid Rafts on WNT/*β*-catenin signaling, we first show their existence in ReNcell VM197 cells and whether they can be disrupted by methyl-*β* cyclodextrin (MbCD) treatment. MbCD is commonly applied to disrupt the formation of lipid rafts by withdrawing cholesterol from the membrane. Previous studies reported an involvement of lipid rafts in the canonical WNT signaling pathway, but these studies were mainly based on detergent resistant membranes (DRM) and applied to proliferating cells, like HEK293 [14–17]. For differentiating cells, however, lipid rafts and their impact on WNT/*β*-catenin signaling have not been documented so far.

**Fig 1:**
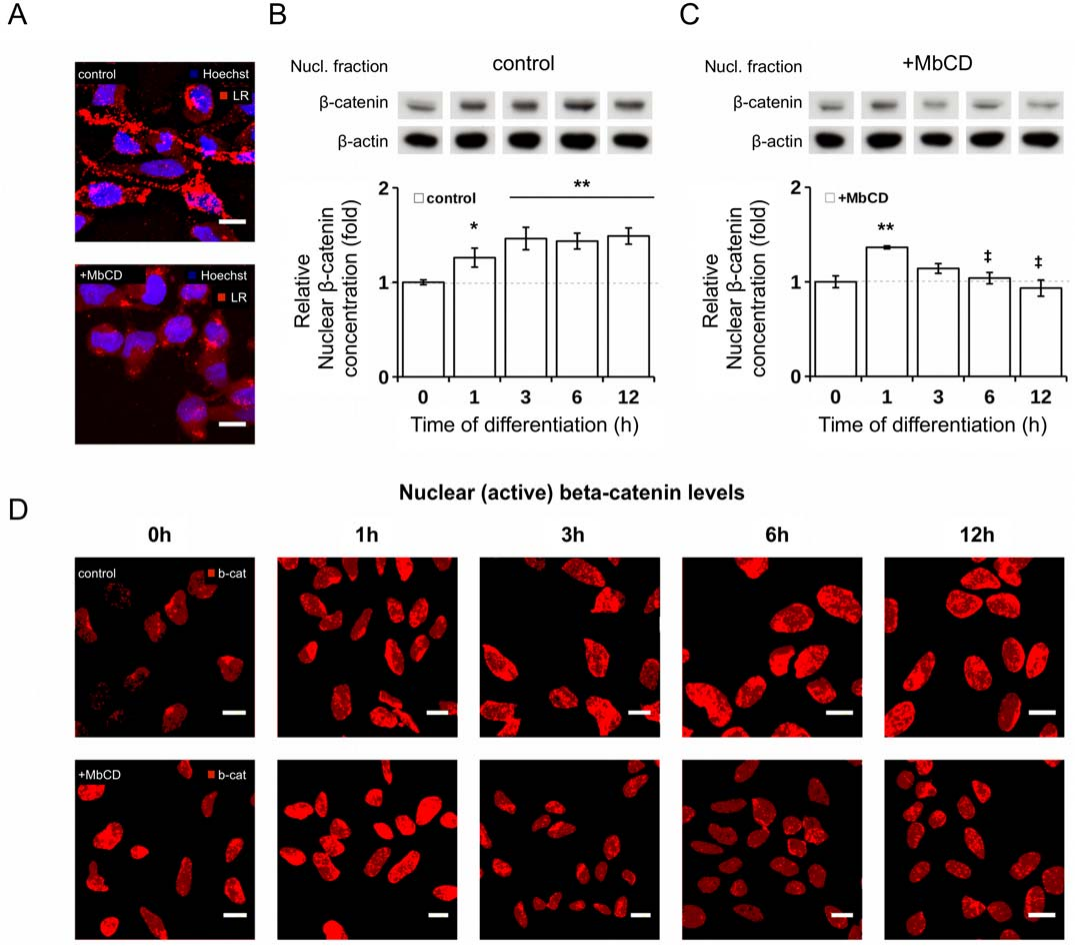
Impact of raft disruption on temporal regulation of nuclear *β*-catenin concentration after induction of differentiation in ReNCell VM197. (A) Confocal microscopy images of Lipid Rafts staining (red) in control (upper row) and raft-deficient, MbCD treated cells (lower row). Cell surface was stained with Vybrant Lipid Raft Labelling kit and nuclei were stained with Hoechst staining. Scale bar 10*μ*m. (B-C) Time-dependent relative concentration levels of nuclear *β*-catenin during differentiation with (C) and without (B) MbCD treatment. Graphs show data of four and three independent experiments for control and MbCD-treated cells, respectively, as mean ± SEM, Student’s t-test (*p < 0.05; **p < 0.01; significant difference from 0h (proliferation); ^‡^p < 0.05; significant difference between control and MbCD treated cells at specific time point), *β*-Actin was used a loading control. (D) Confocal microscopy images of nuclear *β*-catenin signal intensity in control and MbCD treated cells during differentiation confirm western blot data. Cells were labeled with anti-*β*-catenin antibody (red) and Hoechst Nuclei staining. Scale bar = 10*μ*m. For illustration purpose, only the *β*-catenin concentration within the nuclei are shown and other cell compartments, like cytoplasm and membrane are excluded from the view. Please see S2 Fig and S3 Fig for the entire microscopy images from which nuclei sections were extracted.

Indeed, fluorescence microscopy images of ReNcell VM197 cells stained with Vybrant lipid rafts labelling kit confirm the existence of lipid rafts also in human neural progenitor cells (see Fig. 1A). Further, signal intensity of lipid rafts staining is clearly reduced for cells treated with 2mM MbCD in comparison to untreated control cells. Treatment with 2mM MbCD thus successfully disrupts lipid rafts in ReNcell VM197 cells. Also MbCD has little to no effect on the lateral distribution of LRP6 in the membrane. LRP6 staining without application of Lipid Rafts staining shows a homogeneous distribution of LRP6 throughout the entire membrane for both control and MbCD-treated cells (cf. S1 Fig). This is in line with previous studies, that reported no specific partition of LRP6 into Lipid Rafts, but rather a homogeneous distribution among all membrane compartments [14, 15].

#### The impact of lipid raft disruption on *β*-catenin signaling in human neural progenitor cells

To determine the actual impact of lipid rafts on WNT signaling, we treated ReNcell VM197 cells with 2mM methyl-*β* cyclodextrin and measured the nuclear *β*-catenin concentration during early differentiation. Note that cholesterol depletion by MbCD is a concentration dependent and reversible process [33]. To assure a stable and continuous raft inhibition, we thus continuously exposed ReNcell VM197 cells to 2mM MbCD throughout the differentiation. For more details see Material and Methods section. The resulting effects in terms of the nuclear *β*-catenin concentration have been studied qualitatively by fluorescence microscopy and quantitatively by Western Blot.

As a result we register a continuous *β*-catenin signal during differentiation for untreated cells, i.e. for all time points from 1h to 12h the measured nuclear *β*-catenin concentration is significantly higher as compared to proliferating cells (0h) (see Fig. 1B,D). For the MbCD treated cells, however, we observe a significant increase of nuclear *β*-catenin at 1h, but no signal activity after that, i.e. the nuclear *β*-catenin concentration returns to its base line for the remaining time points (3—12 hours) (see Fig. 1C,D). Apparently WNT/*β*-catenin signaling is inhibited by raft disruption after 3 hours of differentiation, but not during the early immediate cell response at 1h.

As demonstrated by earlier and recent studies, the deployment of lipid rafts from the plasma membrane prevents the raft dependent LRP6 phosphorylation and thereby inhibits the WNT induced receptor activation and subsequent signal transduction [15, 17], which could explain the inhibition of WNT/*β*-catenin signaling by MbCD treatment after 3 hours. However, the early immediate activation at 1h in raft deficient cells remains puzzling. As demonstrated, MbCD effectively disrupts lipid rafts in ReNcell VM197 cells (cf. Fig. 1A). Also a delayed raft inhibition cannot be held responsible because MbCD treatment has an immediate effect on the deployment of lipid rafts from the plasma membrane [33]. From this we deduce that lipid rafts are successfully disrupted by MbCD treatment throughout the entire differentiation process and further conclude that, in accordance with previous studies, WNT/*β*-catenin signaling is inhibited by lipid rafts disruption [15, 17]. Though, the early immediate *β*-catenin activation at 1 hour was not affected by MbCD treatment for unknown reasons.

To explore the signaling mechanisms of both, the continuous activation pattern in untreated and in particular the early immediate response in raft-deficient cells, we perform a number of simulation studies based on a validated computational model of WNT signaling we will present in the following.

### A comprehensive model of WNT/*β*-catenin signaling

**Fig 2:**
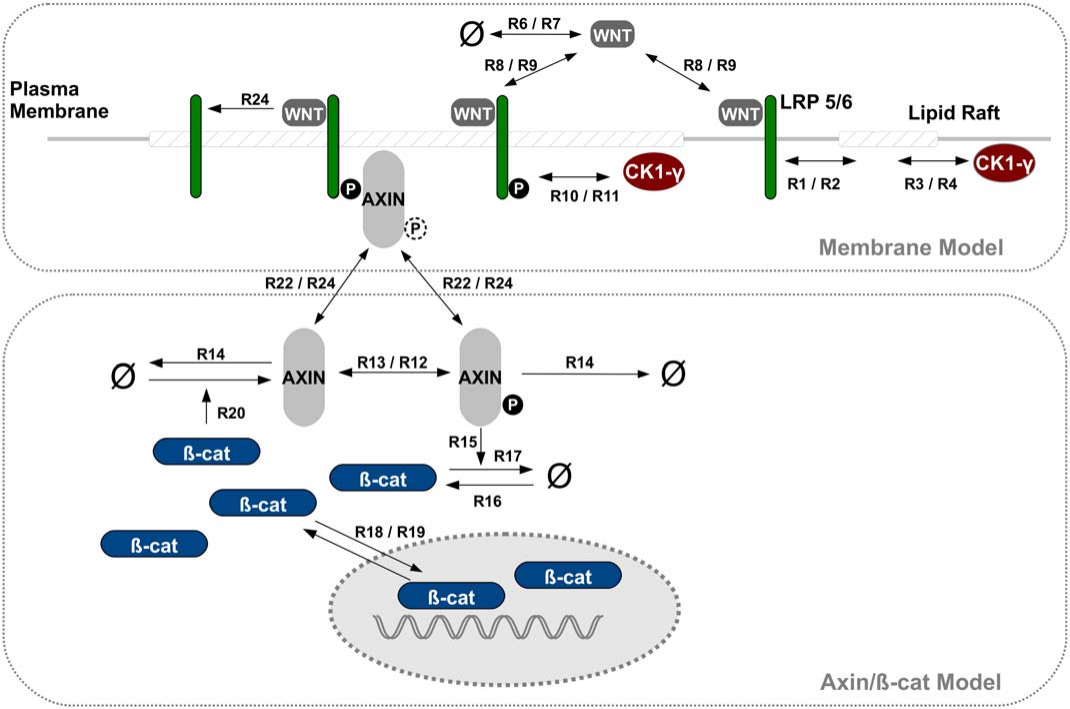
WNT/*β*-catenin model combining membrane and intracellular kinetics. Schematic view of the implemented model combining membrane and intracellular kinetics of WNT/*β*-catenin signaling. In the upper half all membrane-related dynamics included in the model are illustrated. The lower half shows the intracellular processes incorporated in the model, i.e. cytosolic and nuclear dynamics as modeled in [44]. Two-sided arrows indicate reversible reactions. Dashed phosphorylation signs indicate that the depicted protein complex (i.e. AXIN/LRP6) and the corresponding reactions occur independently of the phosphorylation state. The corresponding reaction rate constants are listed in Table 1. For the formal model implementation in ML-Rules see Supporting Information (S3 Text).

Fig. 2 shows a schematic representation of our basic WNT model, i.e. the two main model components of membrane-related LRP6/CK1*γ* and axin/*β*-catenin signaling and their interaction. The model is defined in ML-Rules, a hierarchical, multi-level modeling language [34]. The model is stochastic, multi-compartmental and completely based on mass action kinetics. For a more detailed introduction of ML-Rules and for the implementation of the basic WNT/*β*-catenin model see Supporting Information (S1 Text and S3 Text).

**Table 1:** Parameter Table of the WNT/*β*-catenin model.

**Category/Rule number**	**Parameter**	**Description**	Value	Reference
*Molecule Numbers*				
	WNT	total WNT	220	
	LRP6 (mem)	total membrane-bound LRP6	**4000**	[81]
	CK1y (mem)	total membrane-bound CK1y	*5000*	
	Dvl (cyt)	total cytosolic DVL	*5855*	
	Beta-cat (cyt)	initial cytosolic *β*-catenin	**12989**	[27, 44]
	Beta-cat (nuc)	initial nuclear *β*-catenin	**5282**	[27, 44]
	Axin (cyt)	initial cytosolic AXIN	**252**	[44]
	Axin-P (cyt)	initial cytosolic phosphorylated AXIN	**219**	[44]
*Raft Parameters*				
	R in %	Raft coverage	**25**	[37]
	R_*r*_ (a.u.)	Raft radius	**4**	[37]
	R_*ρ*_	Raft fluidity	*0.1*	
	R_*ϕ*(*LRP*6)_	Raft affinity LRP6	*0.15*	
	R_*ϕ*(*CK*1*y*)_	Raft affinity CK1y	*1*	
	R1	Raft entry of LRP6	*25.12*	
	R3	Raft entry of CK1y	*250.12*	
	R2/4	Raft exit	*25,12*	
*Reaction Rate Constants*				
R6	kWsyn	WNT production	*1.9*	
R7	kWdeg	WNT degradation	*0.27*	
	kWdelay	Delay for WNT production	*90*	
R8	kLWNTBind	LRP6-WNT binding	*100*	
R9	kLWNTUnbind	LRP6-WNT dissociation	*0.1*	
R10	kLphos	Phosphorylation of LRP6 by CK1y	*6.73E1*	
R11	kLdephos	Dephosphorylation of LRP6	*4.7E-2*	
R22	kLAxinBind	LRP6-AXIN association	*5*	
R24	kLAxinUnbind	LRP6-AXIN dissociation	*3E-4*	
R12	kAAp	Basal dephosphorylation of AXIN-P	**0.03**	[44]
R13	kApA	Basal phosphorylation of AXIN	**0.03**	[44]
R14	kAdeg	AXIN degradation	**4.48E-3**	[44]
R20	kAsyn	AXIN synthesis	**4E-4**	[44]
R15	kBetaDegAct	AXIN-driven degradation of *β*-catenin	**2.1E-4**	[44]
R16	kBetaSyn	*β*-catenin synthesis	**600**	[44]
R17	kBetaDeg	basal degradation of *β*-catenin	**1.13E-4**	[27, 44]
R18	kBetaIn	*β*-catenin shuttling into nucleus	**0.0549**	[44, 82]
R19	kBetaOut	*β*-catenin shuttling out of nucleus	**0.135**	[44, 82]

Parameter and reference values of the WNT/*β*-catenin model as depicted in Fig. 2. **Bold:** literature values, *Italics*: fitted values.

#### Model assumptions

In the following we describe certain assumptions we included in our model, either for simplicity or due to a lack of experimental data.

With regard to the membrane compartment, we reduce the representation of the receptor-complex and the signalosome. Accordingly, the FZ-LRP6 receptor complex is only represented by LRP6, such that in our model WNT directly binds to the LRP6 receptor. This simplification is reasonable for canonical WNT signaling, because crucial events, like AXIN binding, mainly depend on LRP6 and its activation through phosphorylation.

We further employ a simplified representation of LRP6 phosphorylation. LRP6 has to be phosphorylated at several phosphorylation sites to recruit and bind AXIN. Thereby the dual phosphorylation of the phoshporylation sites T1479 and S1490 by CK1*γ* and DVL/GSK3*β* is crucial [12, 15, 35]. In our model, we consider solely the interaction between CK1*γ* and LRP6, whereas a detailed representation of DVL mediated unspecific phosphorylation of LRP6 by GSK3*β* is omitted. This assumption is justified by several studies indicating that the LRP6 phosphorylation site targeted by GSK3*β*, S1490, is constitutively phosphorylated and not or only weakly responsive to WNT stimulation, while the phosphorylation of the CK1*γ* specific phosphorylation site, T1479, is clearly induced by WNT stimulation [12, 36].

In addition, we include lipid rafts as individual compartments within the membrane, similar to the nucleus being a single compartment within the cell. The model itself is compartment-based, but for rate calculation we consider the membrane as a two-dimensional layer with lipid rafts being (immobile) circular-shaped entities within the membrane, whose radius and coverage control the rate of receptor-raft collision. In our model we set the radius and number of rafts such that *R*_*A*_ = 25% of the membrane surface is covered by lipid rafts [37]. Membrane bound proteins and receptors may enter and leave individual lipid rafts by diffusion. Note that the mobility inside lipid rafts is reduced. Accordingly the diffusion coefficient of raft-associated receptors is reduced by a constant factor *ρ*. The value of *ρ* controls the extend of receptor aggregation inside lipid rafts [38–40]. In addition to *ρ*, the aggregation also depends on the protein’s specific raft affinity *ϕ*. The value of *ϕ* is mainly determined by the structure and the hydrophobic character of the membrane-bound protein, in particular of its membrane integral domain. This corresponds to the observation, that only a specific set of proteins are accumulated by lipid rafts [41, 42].

In our intracellular model we solely consider AXIN as a condensed representation of the destruction complex disregarding its remaining components, like GSK3*β*, APC amd CK1*α*. This is possible, because AXIN is the main component of the destruction complex and is present in a very low concentration [27]. Although, AXIN has been found to be less rare in mammalian cells than e.g. in Xenopus egg extracts, AXIN is still the rate-limiting component in WNT/*β*-catenin signaling and LRP6-AXIN binding is one of the crucial events for pathway activation [12, 43, 44]. If unbound, the phosphorylation of AXIN directly determines the activation state of the destruction complex, i.e. while unphosphorylated AXIN is inactive, its phosphorylated form is active and promotes the degradation of *β*-catenin. In addition, the destruction complex can be further inhibited by the direct binding of AXIN to phosphorylated LRP6 (p-LRP6), which renders AXIN unavailable for other reactions. Note, that the AXIN/p-LRP6 binding is independent of the phosphorylation state of AXIN.

For the remaining intracellular AXIN/*β*-catenin dynamics, we reuse parts of a previously published AXIN/*β*-catenin model of our group including all relevant parameter values [44]. The AXIN/*β*-catenin model is a simplified and stochastic implementation of the Lee model that contains the most relevant parts to retain the essential dynamics of the full reference model [45]. We further regard the nucleo-cytoplasmic shuffling of *β*-catenin in our model as a simple diffusion process with rate constants based on experimental data, cf. [44].

We further allow two types of WNT stimulation. WNT molecules can either be initially provided (transient stimulation) or continuously synthesized and secreted by the cell. Since WNT is a highly lipophilic protein that is localized at the membrane after its secretion [46, 47], we assume, that released WNT molecules can directly induce the WNT/*β*-catenin signaling at the cell surface in an autocrine manner. Note that in our model we consider only one cell, instead of a heterogeneous cell population. As shown in our aforementioned study, the impact of the cell cycle asynchrony on the average *β*-catenin dynamics in cell populations is negligible [44]. Naturally, in a cell population, the released WNT molecules will most likely induce WNT/*β*-catenin signaling in the neighboring cells as well (paracrine activation).

#### Molecules and interactions

Our basic model, as depicted in Fig. 2 and the model code (S1 Text), is shortly described as follows. Note that, if not stated otherwise, all reactions are reversible, e.g. the rules for WNT binding to LRP6 (R8–9) with rate *kLWNTBind/kLWNTUnbind* relate to the binding and dissociation rate, respectively. LRP6 and CK1*γ* are located in the membrane, both diffusing into and out of Lipid Rafts (rules R1–4). Extracellular WNT binds to LRP6 (R8–9), and subsequently the WNT-LRP6 complex gets phosphorylated by CK1*γ* (R10–11). This reaction is restricted to lipid rafts. The reason for this restriction will be explained in the paragraph “parameter adjustment”. Phosphorylated LRP6 recruits and binds AXIN (R22/24) which is subsequently not available for the destruction complex, i.e. inhibiting the enhanced degradation of *β*-catenin (R15). Thus beta-catenin accumulates and is transported into the nucleus (R18–19). A negative feedback loop is introduced by nuclear *β*-catenin dependent AXIN production (R20). Without WNT stimulation AXIN is subject to frequent autophosphorylation and dephosphorylation (R12–13). In its phosphorylated state, AXIN enhances the degradation of *β*-catenin (R15).

Additionally *β*-catenin, AXIN and WNT are subject to production and degradation processes (R16–17, R14&20, R6–7). Also, the dissociation of the LRP6/AXIN complex (R23–24) is supposed to mimic the recycling of the receptor/protein complex. Consequently in constrast to LRP6 and AXIN, WNT is not released, but consumed in this reaction. The corresponding parameter values for all reaction rate constants are listed in Table 1 including references, if available. For the entire formulation of the model in ML-Rules, see Supporting Information (S4 Text).

#### Parameter Adjustment

Due to the lack of literature values, some parameter values, especially regarding the membrane model, had to be fitted by simulation experiments. The values of the fitted parameters are listed in *italics* in Table 1. First, we adjust the parameters related to the lipid raft/protein interaction, i.e. determine the fraction of LRP6 and CK1*γ* that are associated to lipid rafts. Fortunately, the concentrations for raft associated LRP6 and CK1*γ* have been determined in a previous study [15]. About 30% of LRP6 and 80–85% of CK1*γ* have been found in detergent resistent membranes (DRM). To match these experimentally measured values, we apply different raft affinity values for LPR6 and CK1*γ*. Based on the values in Table 1, the system almost immediately reaches a stable equilibrium with the desired concentration of raft-associated proteins, as depicted in S4 Fig. In addition several recent studies also revealed that CK1*γ* dependent phosphorylation of LRP6 is confined to lipid rafts [15, 17]. We include this finding in our model by restricting the phosphorylation to rafts-associated proteins, i.e. only LRP6 that are located within a lipid raft may be phosphorylated by CK1*γ*. Interestingly, without this constraint we were not able to determine a parameter configuration matching the simulation results to in vitro measurements. This means, the restriction of LRP6 phosphorylation to lipid rafts in the model is not only motivated by the aforementioned studies, but necessary to yield the dynamics observed in vitro.

In the following we fitted the remaining parameter values of the combined intracellular and membrane model against in vitro measurements we derived from human neuronal progenitor cells (ReNcell VM197). More details about the experimental data and in vitro experimentation are described in the previous Section and in the Material and Methods Section respectively. Briefly, we measured the temporal progress of endogenous WNT signaling in terms of nuclear *β*-catenin concentration fold changes during early differentiation in ReNcell VM197 cells. Differentiation of ReNcell VM197 cells is induced solely by growth factor removal and proceeds without any additional external stimulation. The established parameter values of the fitting routine are listed in Table 1. The simulation-based fitting experiment has been specified with SESSL [48]. A short introduction to SESSL is given in the Supporting Information (S1 Text).

As a result of the parameter adjustment, we were able to reproduce the temporal dynamics of nuclear *β*-catenin measured in ReNcell VM197 cells. Before we extensively discuss the simulation results, we first thoroughly validate the model and its current parametrization.

#### Validation of the model

We validated the presented model of WNT/*β*-catenin signaling against independent in-silico and in-vitro data [27, 49]. Thereby, we evaluated how the model reacts on transient and continuous WNT stimulation in comparison to already published data.

**Fig 3:**
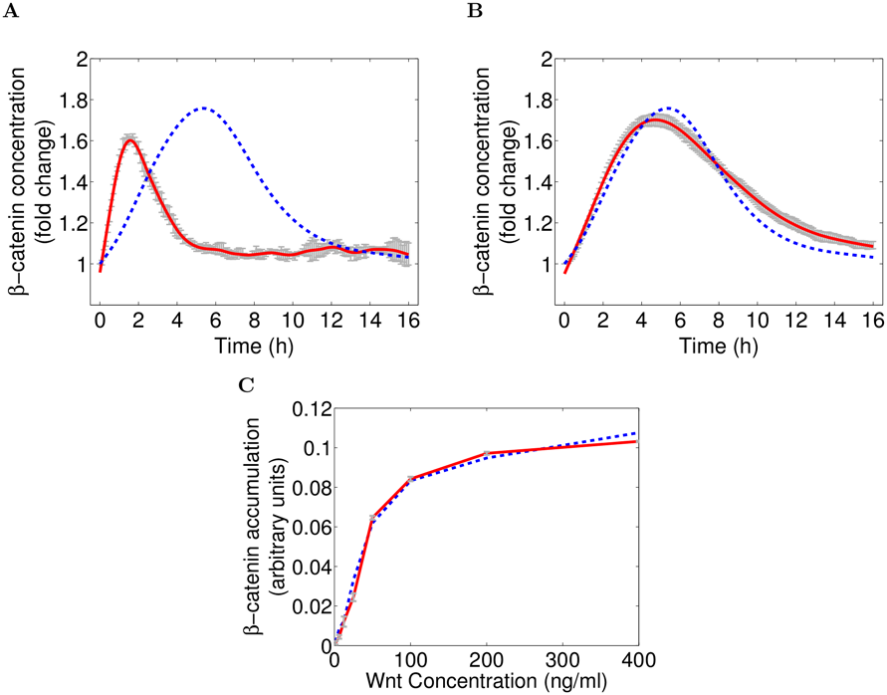
*β*-catenin activation in response to transient and continuous WNT stimuli. (A-B) Comparison of simulation results (*β*-catenin concentration fold change) between the newly derived WNT/*β*-catenin signaling model (red line) and the Lee model (blue, dashed line) [27] in response to a transient WNT stimulus. Without adaptation both models expose a similar excitation level, but the temporal scale differs significantly (A). Adopting the temporal scale of our WNT/*β*-catenin signaling model yields similar simulation results for both models (B). (C) *β*-catenin accumulation after 2 hours of WNT stimulation with varying concentrations, compared between our simulation results (red line) and experimental in-vitro measurements by *Hannoush* (blue line) [49]. Parametrization of the *β*-catenin model is exactly the same as listed in Table 1, despite the WNT production rate (*k*1), which has been parameterized in accordance to the varying WNT stimuli applied by *Hannoush*, cf. Table 2. The simulation results match almost perfectly with the experimental data for all WNT concentrations applied. Note that the in-silico *β*-catenin concentration values are scaled by a linear scaling factor to allow a comparison with the experimentally derived values, that measure the *β*-catenin accumulation based on fluorescence intensities, instead of concentration or fold changes. Simulation results for our model corresponds to mean simulation trajectory (red) with 95% confidence interval (gray error bars).

For the transient stimulation we assume an initial amount of 250 WNT molecules that is degraded over time (see kWdeg in Table 1). This resembles the simulation experiment performed by *Lee et. al.* based on their mathematical model of WNT/*β*-catenin signaling. When comparing the simulation outcome of *Lee et. al.* and our model, it appears that the amplitude or excitation level of the transient signal activity, is similar in both models, but the corresponding temporal resolution differs significantly: In our model the peak of the activation curve (which translates to maximum *β*-catenin concentration) is reached at about 90 minutes and the base line is reached within five hours, while in the Lee model it takes about 5 hours to reach the peak and 16 hours to return to the base line, respectively (cf. Fig. 3A). Apparently, the two models relate to a different temporal scale. However, we can adapt the temporal scale of our model by reducing *all* parameter values by a constant factor. Thereby the system’s kinetics are slowed down, but the inherent system dynamics remain unchanged. To match the temporal level of the Lee model, we apply a constant factor of 2/7. The simulation results with the adapted model are depicted in Fig. 3B and show a good fit between *β*-catenin concentration in our and in the Lee model over the course of time (Fig. 3B). Thus our core model yields the same increase of *β*-catenin concentration in response to a transient WNT stimulus, as predicted by the Lee model when adapting the temporal scale. In this context, we would like to emphasize the rapid differentiation process of ReNcell VM197 cells. This cell line differentiates into neurons and glial cells within 72 hours after growth factor removal, which might explain the faster time scale of our model compared to the Lee model. To model the continuous WNT stimulation, however, we have to compensate the fact, that *in vitro* a single cell is faced with a constant concentration of WNT molecules. This means ligands consumed by the cell (e.g. by receptor binding, endocytosis or unspecific decay) can be immediately replaced by new ones from the bulk solution. This is not the case in our stochastic, single cell model, where we have molecule numbers instead of concentrations. Therefore we apply a production rule for extracellular WNT molecules (modeled as constant flux, R6) with varying rate values according to [49]. To avoid an over saturation of the system, i.e. the number of produced molecules is greater than its consumption, the execution of this *production rule* is restricted to WNT molecule numbers less than a given threshold. This restriction is reversible. Hence, the production of WNT is suspended once the number of WNT molecules exceeds a previously defined value (threshold *ε*), but resumed as soon as the molecule concentration falls below this threshold (cf. rule R6a in S4 Text). For the given validation experiment, the threshold always corresponds to the concentration of WNT molecules tested in the respective simulation run.

**Table 2:** Table of varying WNT stimuli.

**[WNT] ng/ml**	**k1/kWsyn**
1.56	0.3225
6.25	1.29
12.5	2.58
25	5.15
50	10.31
100	20.62
200	41.25
400	82.5

Varying WNT stimuli applied in vitro by *Hannoush* and corresponding input parameter (k1/kWsyn) for model simulations. Concentration values have been recalculated to molecule numbers per available volume (membrane) (details see Text (Paragraph “Validation of the model”)).

Given this slight modification of our model, we run several simulation experiments with the WNT concentrations listed in Table 2 and measured the rate of *β*-catenin accumulation after 2 hours of WNT stimulation [49]. Note, that *Hannoush* measured the accumulation in terms of fluorescence intensities instead of concentration or fold changes. We thus scaled the simulated *β*-catenin concentration values by a linear scaling factor to compare our simulation results with the experimentally derived values. Intriguingly our results (red line) almost perfectly match the experimental data obtained by *Hannoush* (blue line). Regardless of the applied WNT3a concentration, our model always predicts an equivalent *β*-catenin accumulation as obtained *in vitro* (see Fig. 3C). This is underpinned by the fact, that both unscaled data sets—in silico and in vitro—are significantly correlated (*P* = 0.9963, with p-value < 0.001)). To summarize, our WNT/*β*-catenin model, which has been fitted against experimental data retrieved from ReNcell VM197 cells solely, is capable of exactly reproducing *β*-catenin kinetics reported for different cell types and stimuli (transient and continuous WNT3a stimulation) [27, 49]. Consequently our WNT/*β*-catenin model is not only in agreement with data published earlier, but conclusions about WNT/*β*-catenin signaling drawn from ReNcell VM197 cells do not appear to be cell line specific and, hence, seem generally applicable.

#### Hidden biphasic activation pattern

Before we analyze the effect of lipid rafts disruption on canonical WNT signaling and execute the corresponding simulations, let us take a closer look at the simulation results achieved so far. As previously mentioned, all unknown parameter values were derived by fitting the model to our in vitro measurements of endogenous WNT signaling in ReNcell VM197 cells.

**Fig 4:**
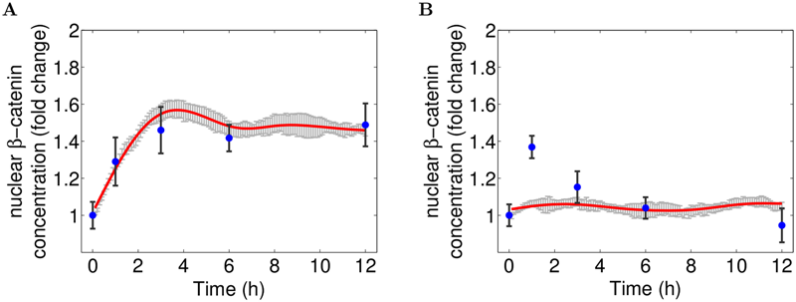
Experimental data vs. Simulation results. Nuclear *β*-catenin concentration fold changes in comparison between experimental data and the validated WNT/*β*-catenin model. The simulation result (red) of the WNT/*β*-catenin model (cf. Fig. 2, parametrized according to Table 1) matches all experimental values (blue) in untreated control cells (A). Though, in its current state it is not capable of reproducing the immediate early *β*-catenin activation in raft-deficient cells (B). Simulation results correspond to the mean simulation trajectory (red) with 95% confidence interval (gray error bars).

Considering the input parameter values that are required to reproduce our experimental data, it appears that only a model parametrized with an initial amount of WNT molecules (*nWNT = 90*) and a constant WNT synthesis rate (*kWsyn = 1.9*) after a certain delay of 90 minutes yields the desired simulation result. This detail is of great importance, as it suggests that *β*-catenin accumulation is caused by two different *WNT stimuli*—an initial, transient trigger and a continuous, autocrine signal mechanism. It is the combination of these two WNT stimuli, that allows the cell to first generate an immediate response to the perturbation (removal of growth factor) and in the following to keep the activation on a constant, but moderately incremented level (cf. Fig. 4A). With regard to the continuous autocrine signal, our findings are in line with a previous study of our group, where we used a simplified computational model to provide evidence for the self-induced autocrine/paracrine WNT signaling in hNPCs [44]. Thus, our experimental and computational studies underpin our in silico derived hypothesis. In addition, several other studies describe continuous autocrine canonical WNT signaling in the context of neural stem cells [50] and cancer [51, 52].

In contrast, it is not entirely clear where the immediate, transient WNT stimulus might originate from. Possible explanations are that cytosolic vesicles fuse with the membrane in order to spontaneously release a certain amount of WNT molecules [47], and that the initial stimulus is a direct result of crosstalk with growth factor pathways [53].

#### WNT independent signaling mechanism triggers early immediate *β*-catenin activation

For raft-deficient cells, the simulation trajectory does not show any signal intensity, i.e. the nuclear *β*-catenin concentration stays at its base line (cf. Fig. 4B). This behaviour seems only natural, because in our model the MbCD treatment translates to a complete removal of lipid rafts, which in turn prevents the raft-dependent LRP6 phosphorylation by CK1*γ* in response to a WNT stimulus [15]. Thus WNT molecules may still bind, but the receptor activation and hence the transduction of the extracellular WNT signal is blocked. As a result we would expect a complete inhibition of WNT signaling when disturbing lipid rafts, as predicted by our model.

Though, western blot as well as fluorescence microscopy data indicate a significant increase of nuclear *β*-catenin at one hour of differentiation for raft deficient cells (see Fig. 1C-D). This implies a successful activation of WNT/*β*-catenin signaling for this time point, despite lipid rafts disruption. As the deployment of lipid rafts primarily affects membrane-related processes, like the WNT-induced phosphorylation of LRP6, it stands to reason that the activation of *β*-catenin signaling in raft deficient cells is likely caused by an alternative WNT/LRP6-independent signaling mechanism. Pursuing this line of thought further: What if the early immediate cell response in raft-deficient *and* control cells was triggered by one and the same signaling mechanism, that is completely independent of membrane-related processes and therefore unaffected by raft disruption? In such a scenario, we would find characteristic upstream WNT signaling components already being inactive in untreated control cells with simultaneous (nuclear) *β*-catenin accumulation. Indeed, earlier studies on the same cell line, provide experimental data, that show these dynamics for the early immediate cell response in untreated ReNcell VM197 cells: p-LRP6 was found to be NOT significantly increased during the early time points (0–3 hours), while *β*-catenin shows the ascribed transient activation (cf. [8]). At the same time, the positive control confirmed that cells are responsive to WNT stimulation, i.e. transient WNT3a treatment yields a significant increase of p-LRP6 within the membrane. This means in the undisturbed case, *β*-catenin stabilization is observed, even though upstream WNT signaling components are inactive, but functional. This apparent contradiction clearly underlines our hypothesis of wnt-independent signaling stabilizing and translocating *β*-catenin into the nucleus. On the one hand, this result corroborates our hypothesis that lipid raft dependent, autocrine WNT signaling induces the continuous *β*-catenin activation. On the other hand our results raise the question what mechanism triggers the early immediate cell response at 1 hours?

### Endogenous ROS signaling as potential trigger for *β*-catenin signaling

We demonstrated, that the combined membrane and axin/*β*-catenin model captures relevant processes of canonical WNT signaling and is able to predict the WNT/*β*-catenin dynamics in response to arbitrary WNT stimuli of untreated cells with undisturbed lipid rafts. Though, the model is not capable of reproducing the transient activation in raft-deficient cells (see Fig. 4B). To predict this apparently WNT-independent signal, the present WNT/*β*-catenin model has to be extended by a presumingly intracellular mechanism.

In a recent study with the same cell line, we uncovered an endogenous, WNT-independent activation of WNT/*β*-catenin signaling through reactive oxygen species (ROS) in response to initiation of differentiation through growth-factor removal [21]. Thereby an increase of the intracellular ROS level releases the redox-sensitive binding between NRX and DVL, hence promoting a DVL-mediated stimulation of the downstream WNT/*β*-catenin signal transduction, which eventually leads to the well known *β*-catenin accumulation in the nucleus. In fact, several experimental studies demonstrating a redox-dependent activation of WNT/*β*-catenin signaling have emerged recently. *Funato et. al.* reported a robust activation in response to exogenous ROS stimulation in proliferating cells [25], while *Love et. al.* showed that injury-induced ROS is required to activate WNT/*β*-catenin pathway in the context of cell regeneration [26]. Whereas extensive ROS stimulation may cause oxidative stress and cell damage, it is meanwhile well accepted, that ROS can also act as intracellular messenger inducing redox-sensitive signal transductions when present at physiological concentrations [54]. To evaluate, whether an interplay between redox- and lipid raft dependent, autocrine WNT/*β*-catenin activation is a suitable hypothesis to explain our data, we extend our model with a redox-dependent/*β*-catenin pathway. Since quantitative experimental data is rarely available, we base our model upon the findings of *Funato et. al.* and our own recent experimental results [21].

**Fig 5:**
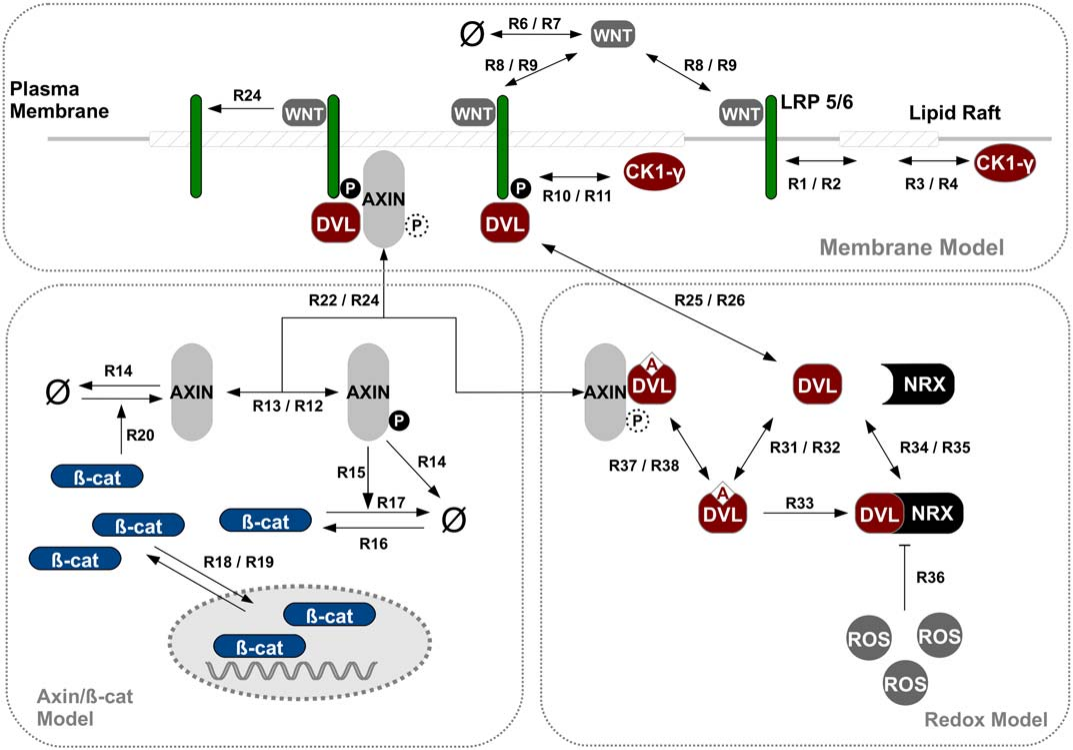
Extended WNT/*β*-catenin model including ROS/*β*-catenin signaling. Schematic view of the extended WNT/*β*-catenin model illustrating the potential interplay between WNT/*β*-catenin- and DVL-mediated ROS/*β*-catenin signaling. In addition to the previous model (cf. Fig. 2), the newly introduced WNT-independent redox-signaling is depicted in the lower right. Two-sided arrows indicate reversible reactions. Dashed phosphorylation signs indicate that the depicted protein complex (i.e. AXIN/DVL and AXIN/DVL/LRP6) and the corresponding reactions occur independently of the phosphorylation state. The corresponding reaction rate constants are listed in Table 1 and Table 3. The entire model implementation in ML-Rules can be found in the Supporting Information (S4 Text).

As depicted in Fig. 5 we extend the given model by a new, redox-dependent model component. For a complete implementation of the extended model see Supporting Information (S9). According to the aforementioned studies, ROS molecules release the redox-sensitive binding of DVL and Nucleoredoxin (NRX), leading to a spontaneous increase in cytosolic DVL concentration (R36). Due to the property of DVL to self-associate in a reversible and concentration-dependent manner (R31–R33), DVL forms self-assemblies that serve as dynamic recruitment platform for AXIN [55, 56](R37/R38). DVL-bound AXIN is not available for the destruction complex, hence *β*-catenin can accumulate and translocate into the nucleus. The spontaneous release of DVL through ROS obviously mimics an overexpression of DVL, which has been demonstrated to trigger WNT/*β*-catenin signaling, bypassing the requirement for WNT ligands [25, 55, 57].

**Table 3:** Parameter Table of the extended ROS/*β*-catenin model component.

**Category/Rule number**	**Parameter**	**Description**	**Reference Value**
*Molecule Numbers*			
	Wnt	total WNT	0
	ROS	total initial ROS	10000
	Dvl (cyt)	unbound cytosolic DVL	855
	Nrx	unbound cytosolic NRX	18
	DvlNrx	cytosolic DVL bound to NRX	36200
*Reaction Rate Constants*			
R6	kWsyn	WNT production	1.9
R7	kWdeg	WNT degradation	0.27
	kWdelay	Delay for WNT production	90
R25	kLDvlBind	LRP6-DVL association	2.8E4
R26	kLDvlUnbind	LRP6-DVL dissociation	3.5E-4
R29 (not shown)	kNrxRos	ROS oxidation of NRX	5E2
R30 (not shown)	kNrxNo	NRX reduction	2E-2
R31	kDvlSponAgg	Aggregation of DVL	5E-4
R32	kDvldisAgg	Dissociation of DVL	0.65
R34	kDvlNrxBind	DVL-NRX Association	22.5
R35	kDvlNrxUnbind	DVL-NRX Dissociation	2.3E-2
R36	kDvlNrxRos	ROS oxidation of NRX forcing release of DVL	3.2E2
R37	kDvlAxinBind	DVL-AXIN Association	0.075
R38	kDvlAxinUnbind	DVL-AXIN Dissociation	6.8E-2

Parameter and reference values of the DVL-mediated ROS/*β*-catenin signaling model as depicted in Fig. 5. The remaining model parameter values listed in Table 1 are kept fixed.

**Fig 6:**
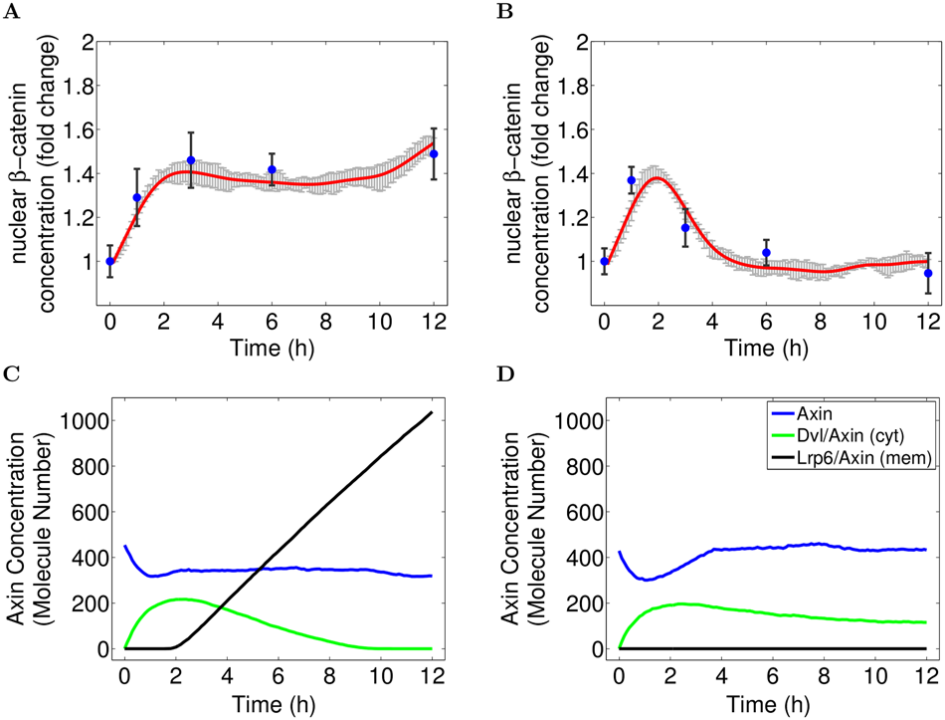
Experimental data vs. Simulation results. (A-B) Nuclear *β*-catenin concentration fold changes in comparison between experimental data and the extended WNT/ROS-*β*-catenin model. The simulation result (red) of the extended WNT/ROS-*β*-catenin model (cf. Fig. 5, parametrized according to Table 1 and Table 3) match all experimental values (blue) in untreated control (A) and raft deficient cells (B). Simulation results correspond to the mean simulation trajectory (red) with 95% confidence interval (gray error bars). (C-D) AXIN concentration in comparison between bound (to DVL or LRP6) and unbound state. Simulation mean trajectories of AXIN in its bound and unbound states for untreated (C) and raft deficient cells (D).

After successful calibration we connect the ROS/*β*-catenin model with the model presented in the previous section. To initiate ROS/*β*-catenin signaling, we introduce a transient ROS signal at the beginning of differentiation [21]. This corresponds to the significant increase of endogenous ROS levels measured in ReNcell VM197 human progenitor cells. Indeed, the extended model is now able to reproduce the immediate *β*-catenin activation in raft deficient cells as well as the kinetics in untreated cells (cf. Fig. 6).

Please note that, all parameter values, but the coverage of rafts (25% and 0), are exactly the same for the simulation of control and raft-deficient cells. For both simulation experiments the model is parameterized with a transient ROS signal and a delayed, constant WNT production. Thus in contrast to the previous model configuration we replaced the initial amount of WNT molecules with a onetime release of ROS molecules (*nRos* = 10000) in response to growth factor removal. All other remaining parameter values of our earlier model remain the same, in particular, the delayed (90min) and constant WNT production (*kWsyn* = 1.9). The necessity to include such a delay can be explained by inspecting our results more closely: Note, that the increase of *β*-catenin concentration during the immediate early response (1h) is not significantly different between control and raft deficient cells. If WNT signaling was directly activated after induction of differentiation, the signal at 1 hours would add up with the *β*-catenin activation induced by ROS, hence most likely be significantly higher in control than in raft deficient cells. As this is not the case, we conclude, that the described autocrine, raft-dependent WNT signaling can only be initiated after a certain delay. However, this also implies that the signal after one hour is entirely based upon WNT/LRP6 independent mechanisms like the presented redox-dependent DVL/*β*-catenin pathway.

This becomes even more evident, when considering the localization and binding state of AXIN during signaling (cf. Fig. 6C). While unbound AXIN acts as inhibitor of WNT signaling, in place of the complete destruction complex, the (reversible) binding states of AXIN to DVL and membrane-bound LRP6 relate to the two previously described mechanisms for activating *β*-catenin signaling: During the first two hours, *β*-catenin activation solely results from DVL/AXIN binding, i.e. the redox-dependent DVL/*β*-catenin pathway. Only after that, AXIN starts getting recruited to the membrane and bound by the activated LRP6 receptor complex. This process is driven by the auto-/paracrine WNT signaling, which, in the long run, replaces the transient redox-dependent DVL/*β*-catenin pathway, such that AXIN is eventually only bound to LRP6. Note, that due to negative feedback, the elevated concentration of nuclear *β*-catenin enhances the synthesis of AXIN. As a result, in the long run, the binding of AXIN to LRP6 yields an unrestrained linear increase of LRP6/AXIN in control cells for late time points. This indicates that additional mechanisms, like endocytosis and recycling, are required to maintain the continuous auto-/paracrine WNT-signaling for a longer period of time (cf. Conclusion and Outlook).

In summary, our simulation results suggest a two-fold activation mechanism that drives the early differentiation process in human progenitor cells. Accordingly, the cellular response upon differentiation induction through growth-factor removal is characterized by an immediate, transient response through redox-dependent DVL signaling, followed by a constant, auto-or paracrine WNT signaling in a raft-dependent manner.

#### DVL as a concentration-dependent dual signal transducer

We would like to emphasize the dual role that DVL, a central component of both, canonical and non-canonical WNT signaling, plays in this context [57]. On the one hand, DVL is required for the phosphorylation and accumulation of LRP6 and is thus continuously recruited to the membrane in response to WNT stimulation [10, 16, 55]. On the other hand, DVL itself acts as an independent transducer for *β*-catenin signaling in a redox dependent manner, independent of WNT molecules. Obviously the function of DVL is characterized by a highly concentration dependent mechanism.

In the inactive state DVL is primarily bound by NRX [25]. The remaining fraction of unbound DVL is too small to initiate self-aggregation, but sufficiently large to support and enhance WNT-induced receptor activation at the membrane. In fact, this process is enhanced by the localization of LRP6 and CK1*γ* in lipid rafts, which allows a local, density-dependent activation despite the low concentration of unbound DVL [58].

The redox-sensitive release of DVL from NRX in response to the transient ROS signal, however, results in a spontaneous increase of the cytosolic DVL concentration. As a result DVL immediately gets activated by forming self-aggregates, that provide high affinity binding sites for cytosolic AXIN [55] (cf. Fig. 6 C&D). The binding of AXIN by aggregated DVL in turn inhibits the destruction complex, hence activating *β*-catenin signaling. Due to the dynamic nature of DVL aggregates, i.e., their association and disassociation, the *β*-catenin activation is reversible: as soon as the DVL concentration falls below a certain threshold, e.g. by NRX rebinding, AXIN-DVL binding and thus *β*-catenin signaling is inhibited again. As a result, the nuclear *β*-catenin concentration returns to its base-line, as illustrated in Fig. 6B.

To summarize, based on our computational model, we demonstrated, that DVL may either act as amplifier or as direct inducer of canonical WNT signaling. Thereby the state of activity is determined by the concentration and localization of DVL, i.e. low concentrated, membrane-associated DVL amplifies WNT-induced LRP6 receptor activation and signalosome formation, whereas high concentrated DVL directly induces *β*-catenin signaling, e.g. in response to a ROS stimulus. This is in line with a number of in vitro studies, that elucidate the role of DVL during WNT/*β*-catenin signaling [25, 55, 56].

##### Increased ROS production in response to initiation of differentiation is independent of raft disruption

Our simulation studies confirm that the presented model of combined redox and raft-dependent wnt signaling provides a sustained explanation to our experimental data. However, redox signaling and lipid rafts are closely related to each other, since major components of redox signaling mechanism are found to be raft-associated, like NADPH oxidase, superoxide dismutase and Catalase [59–61]. Accordingly, we have to re-evaluate our experimental data, as MbCD treatment may have an additional impact on ROS signaling and might even induce the early immediate response in raft-deficient cells. To test whether the proposed ROS signaling mechanism is independent of the MbCD treatment, we analyzed the mitochondrial ROS (mito-ROS) production in control and raft deficient ReNcell VM197 cells during proliferation and during the early hours of differentiation. To monitor the mito-ROS metabolism we apply MitoTracker Red according to [62]. More details are described in the Material and Methods section.

**Fig 7:**
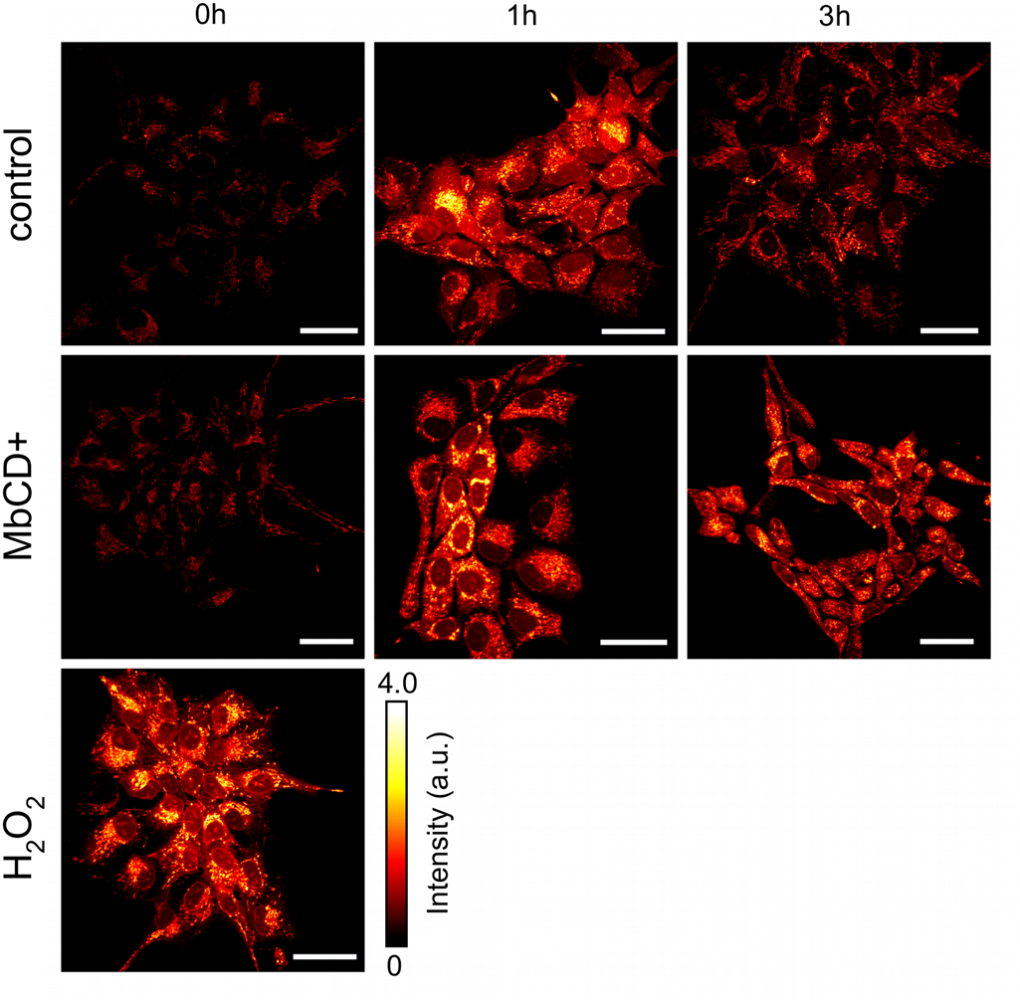
Confocal Microscopy of mitochondrial ROS level. Confocal microscopy of mito-ROS levels for untreated (control) cells and MbCD-treated cells. Proliferating cells have been treated with H_2_O_2_ as positive control and MbCD for 1 hour (first column). 1 hour after induction of differentiation cells show a marked increase of mito-ROS levels, and subsequent decrease after 3 hours. Images further confirm that neither proliferating nor differentiating cells are subject to crucial changes in mitochondrial ROS level due to raft disruption through MbCD treatment. Scale bar 20 *μ*m.

In proliferating state, control and raft-deficient cells show no detectable changes in the mito-ROS level, whereas H_2_O_2_ stimulation results in a significant increase (cf. Fig. 7). Accordingly ROS metabolism is not induced or promoted by MbCD treatment in proliferating cells. After one hour of differentiation we register a transient, marked increase of mito-ROS production in differentiating cells compared to proliferating cells, that is in accordance with the data reported in [21]. The transient increase of mito-ROS production occurs in untreated control as well as in MbCD treated cells (cf. Fig. 7). After three hours, we detect a decrease in the mito-ROS metabolism, that is slightly more pronounced in control than in raft-deficient cells. Apparently MbCD treatment alters the mito-ROS metabolism, but only after three hours of differentiation, whereas the changes in the mitochondrial ROS metabolism in direct response to induction of differentiation occur independently of MbCD treatment. The increased mito-ROS metabolism at three hours likely results from MbCD attenuating the antioxidant system by disrupting its raft associated components, like NADPH oxidase or superoxide dismutase. Consequently, MbCD treatment does not promote the activation of mito-ROS metabolism in response to the induction of differentiation (as described in [21]), but hampers the subsequent elimination of the generated ROS.

However, the increased ROS level at three hours has no apparent effect on *β*-catenin signaling. While mito-ROS metabolism is still increased after three hours in raft-deficient cells (cf. Fig. 7), the nuclear *β*-catenin concentration is returning to its base-line already (cf. Fig. 1C,D). In fact, this insight further corroborates our hypothesis of a biphasic activation pattern, where redox-dependent DVL/*β*-catenin signaling is only active during the early immediate response (1h), while the subsequent continuous *β*-catenin accumulation results from an autocrine/paracrine, raft-dependent WNT/*β*-catenin signaling mechanism (3–12h) (cf. Fig. 6).

### Conclusion and Outlook

In a combined in-vitro and in-silico approach we find strong evidence, that cell fate commitment in human neural progenitor cells is driven by two distinct *β*-catenin signaling mechanisms. According to our simulation results, only a concisely regulated interplay between redox-dependent and self-induced auto-/paracrine WNT signaling can explain the nuclear *β*-catenin dynamics observed experimentally during the initial phase of differentiation:

In response to growth factor removal, a transient increase of the intracellular ROS level activates DVL in a redox-dependent manner. While DVL is primarily bound by NRX in the inactive state, ROS release the redox-sensitive association between NRX and Dishevelled (DVL). This leads to a spontanous increase of unbound DVL molecules, which immediately get activated by forming self-aggregates. Activated DVL subsequently stimulates downstream signaling components causing an immediate transient *β*-catenin signal [25, 55]. After a certain delay, a yet unknown mechanism triggers a continuous production of WNT molecules, which results in a stable activation of WNT/*β*-catenin pathway by auto-/paracrine signaling. The resulting continuous WNT signal is raft-dependent, i.e. the disruption of rafts completely inhibits the signal transduction. Recent studies show that both WNT- and ROS-induced *β*-catenin signaling pathways, are essential positive regulators for the neuronal differentiation as the inhibition of either one significantly reduces the neuronal yield [5, 21].

In addition, we also provide a comprehensive model of WNT/*β*-catenin signaling that for the first time combines intracellular and membrane-related processes including lipid rafts dynamics. Its predictive ability has been demonstrated under a wide range of varying conditions for in-vitro as well as in-silico reference data sets. However, we are well aware, that our model is a simplified representation of WNT/*β*-catenin signaling. As for instance, it does not include any endocytotic processes, like recycling or the sequestration of the destruction complex inside multivesicular endosomes as currently discussed [63, 64]. Though our model does neither contradict nor exclude these hypotheses. Instead we concentrate on the fact, that phosphorylation of LRP6 is a raft-dependent process being crucial for canonical WNT/*β*-catenin signaling as demonstrated by [15] and our investigations. LRP6 phoshporylation is a prerequisite for WNT-mediated endocytosis [14, 63]. The reversible binding of AXIN to activated LRP6, as described in our model is sufficient to accurately predict and reproduce in-silico and in-vitro measurements under varying conditions. However, it is one among many possible mechanisms preceeding LRP6 phosphorylation.

We’d like to emphasize that the model is based on ML-Rules, a multilevel, rule-based modelling language, that facilitates the extension and modification of the model. With regard to the previously mentioned endocytotic processes, endosomes and multivesicular bodies (MVB) may thus be effortlessly included in the model in terms of dynamic, cytosolic compartments. The presented model may thus serve as starting point to further investigate and evaluate current hypotheses referring to WNT/*β*-catenin signaling, like the role of raft-dependent and independent endocytosis [14, 15, 63, 65, 66], the multiple functions of DVL in canonical and non-canonical WNT pathways (crosstalk) [57], or the targeting of WNT molecules through lipid modifications [46, 67].

## Materials and methods

### Wet lab

#### Culture of neural progenitor cells and lipid rafts Disruption

Our experimental results are retrieved from ReNcell VM 197 cells—a cell line, that is derived from the ventral midbrain of a 10-week-old human fetus and immortalized by retroviral transduction with v-Myc oncogene (ReNeuron Ltd, Guildford, UK). VM cells were cultivated according to the protocol described previously [68]. Briefly, cells were cultured in laminin coated cell culture flasks and maintained at 37°C with 5% in media containing DMEM/F12 supplemented with B27 media supplement, glutamine, heparin sodium salt and gentamycin (Invitrogen, Karlsruhe, Germany). Cells were kept in proliferative state by applying 10 ng/mL basic fibroblast growth factor (bFGF, Invitrogen) and 20 ng/mL epidermal growth factor (EGF, Sigma-Aldrich, Steinheim, Germany). Every three to four days the cells were passaged, i.e. when a confluency reached ∼ 80%. Differentiation was initiated at a confluence of ∼ 70% according to a standard differentiation protocol, i.e. cells were washed with HBSS, and new medium without growth factors EGF and bFGF was added [69]. For the continuous lipid rafts disruption troughout differentiation 2mM M-*β*-cyclodextrin (MbCD) was added to the differentiation medium. To exclude potential side effects caused by the MbCD treatment, proliferating cells were also treated 30 minutes in advance of fixation (Immunocytochemistry) or lysis (Western Blot).

#### Fixation and immunostaining for fluorescence microscopy

Before fixation, lipid rafts were labeled with Vybrant lipid rafts labeling kit (Invitrogen). Cells cultured on coverslips were incubated with 0.5mM fluorescent Cholera Toxin B-Subunit (CT-B, Alexa 594) for 10 minutes at 4°C. After washing with PBS, cells were treated with anti-CT-B antibody (dilution 1:200) for another 10 minutes at 4°C. In the following fixation and immunofluorescence staining was performed as described previously [70]. Accordingly, cells were washed with PBS and fixed with 4% paraformaldehyde for 20 min (Sigma-Aldrich). To reduce non-specific binding, cells were treated with 1% gelatin. First, cells were labeled with rabbit anti-LRP6 (Santa Cruz, dilution 1:150) and subsequently incubated with Alexa Fluor 488 (Invitrogen, dilution 1:300). Afterwards, cell membranes were permeabilised with 0.2% Triton X-100 (Sigma-Aldrich) followed by labelling with mouse anti-active-*β*-catenin (Millipore, dilution 1:250) and subsequent incubation with Alexa Fluor 647-conjugated anti-mouse secondary antibody (Invitrogen, dilution 1:300) and Hoechst for nuclei staining (Sigma-Aldrich, dilution 1:1000). Finally, cells were mounted on microscope slides using ProLong Gold antifade reagent (Invitrogen).

#### Mitochondrial ROS Level

For detection of intracellular ROS levels, proliferating cells were incubated with 50nM Mitotracker Red CMXRos (Invitrogen) for 40min. According to [62] the dye strongly accumulates in mitochondria which results in fluorescence quenching. A change in mito-ROS production then induces a dye release leading to a reduction of the quenching with simultaneous rise in the fluorescence. Subsequently, cells were induced to differentiate in the absence or presence of 2 mM cyclodextrine (Sigma-Aldrich). To exclude a ROS-stimulating effect during proliferation, proliferating cells were also treated with 2mM cyclodextrine. The ROS-increasing agent hydrogen peroxide (2mM, Sigma-Aldrich) was used as positive control. Fluorescence was analyzed by confocal microscopy using Nikon A1 confocal imaging system with a 60×/NA 1.4 oil objectives (Nikon, Tokyo, Japan).

#### Western blotting

Protein concentration was determined by Western blotting. Briefly cells cultured were washed twice with phosphate-buffered saline (PBS) and lysed in 29 sodium dodecyl sulfate (SDS) sample buffer followed by sub-cellular fractionation. Cell fraction lysates were separated by SDS-polyacryl-amide gel electrophoresis (PAGE) using a 10% SDS polyacrylamide gel and proteins were transferred onto nitrocellulose membrane by electro blotting. For time-dependent *β*-catenin expression, the following anti-bodies were used: primary antibodies: mouse anti-*β*-catenin (Santa Cruz, dilution 1:1000), anti-*β*-actin (Delta Biolabs, dilution 1:10000), secondary antibodies: Anti-rabbit IgG (Cat. A9169; Sigma-Aldrich, dilution 1: 80 000) and anti-mouse IgG (Cat. NA931V; GE Healthcare, Freiburg, Germany, dilution 1: 10 000) antibodies conjugated with horseradish peroxidase were used and bound antibodies were detected with ECL Western blot detection reagent (GE Healthcare). Membranes were exposed to light-sensitive film and quantified by IMAGEJ software.

#### Hierarchical modeling with ML-Rules

Our model is defined in ML-Rules, a multi-level, rule-based modeling language [34]. Rule-based modeling languages use the notations of chemical equations to describe cell biological systems. Thereby, the state of the model is represented by chemical solutions, i.e. mappings from species to concentrations or discrete numbers, while the transitions between different model states are defined in terms of reactions. For execution, a well defined semantic translates the model into its corresponding mathematical definition, e.g. ordinary differential equations (ODEs) or stochastic processes [71, 72].

Rule-based approaches further benefit from the possibility of describing different molecule states (like phosphorylation states or binding sites) in terms of attributes. This allows to define rules with reaction patterns, where a single rule represents a set of multiple reactions, depending on the attribute values of the species [34, 72, 73]. Thereby the size of the model can be significantly reduced, because a reaction network can be defined in terms of schematic rules instead of enlisting all possible combinations of species and reactions. For a comprehensive review of rule-based modeling the interested reader is referred to [74].

The semantics of ML-Rules is based on continuous time Markov chains (CTMC). ML-Rules models are executed by stochastic, discrete event execution algorithms [75]. All entities are expressed in terms of concrete numbers, like molecules, compartments or cells, instead of concentrations. In our model stochastic events play a crucial role due to the comparatively low molecule number of the key player AXIN. In this setting, a deterministic ODE based execution might miss important dynamics as has been shown in [44]: in comparison to the ODE based execution, the stochastic execution revealed artifacts in simulating *β*-catenin signaling within hNPCs-cells if adopting the very low AXIN concentration as given by [27]. Therefore in [44], a still comparatively low but ∼ 10 times higher number of AXIN molecules was determined as more realistic for hNPCs, a result which was later confirmed for various mammal cells by wet-lab studies [76]. The implemented WNT/*β*-catenin signaling model makes extensive use of rule-schemata provided by the ML-Rules syntax. This is necessary, since the model contains several hierarchical levels as well as protein specific binding and phosphorylation states, that are in particular necessary for the representation of the signalosome. Accordingly, in our model the central component of the signalosome, LRP6, is attributed with four different attributes: diffusion rate, raft affinity, phosphorylation state and binding state. Further individual LRP6 receptors continuously diffuse between membrane and raft regions according to its raft affinity value. Using a non-attributed modeling formalism, these states had to be represented as individual species and the respective reactions had to be considered separately, which would significantly increase the complexity of the model in terms of species/reactants and reactions. Further we’d like to emphasize that ML-Rules allows an easy and straight forward extension of the presented model. As discussed in the outlook, endocytosis and multi-vesicular body handling can be included in the model similar to lipid rafts, i.e. as dynamic, cytosolic compartments. A more detailed description of the model specification in ML-Rules and the corresponding specification of the simulation experiments is given as Supporting Information (S1 Text). For a thorough introduction to the general ML-Rules modeling formalism, the interested reader is referred to [34].

#### In-silico experiments

ML-Rules is implemented on top of the modeling and simulation framework JAMES II [77]. JAMES II is implemented in Java and provides various plug-ins to realize complex simulation experiments, e.g., for parameter optimization, sensitivity analysis, and output data storage [78]. In our experiments, we used the approximative *τ*-leaping simulator for ML-Rules [79] to speed up the simulation. We set up most experiments with the domain-specific language SESSL [48]. SESSL is based on the Scala programming language [80] and allows to concisely specify JAMES II experiments. For a description of two typical experiment setups (a parameter scan and an optimization experiment), that illustrate the specification of simulation experiments in SESSL, see Supporting Information (S1 Text). To reproduce our experiment, please find a sandbox to simulate ML-Rules models and a setup to execute SESSL experiments at http://wwwmosi.informatik.uni-rostock.de/wnt-model-experiments-sessl. The sandbox also contains a user manual to the modeling formalism ML-Rules, describing its concrete syntax and illustrating how ML-Rules can be used for modeling various biochemical and multi-level systems.

## Supporting Information

S1 Fig LRP6 distribution. Confocal microscopy images of LRP6 staining (no Lipid Rafts staining) in proliferating and early differentiating cells. The first row shows untreated (control) cells, while cells depicted in the lower row are treated with 2mM MbCD. Scale bar 10*μ*m
(TIF)

S2 Fig Beta-catenin levels in control cells. Microscopy images depicting beta-catenin staining (red) during early differentiation (0–12 hours) in non-treated ReNcell VM197 control cells. The first row shows the entire cells with beta-catenin (red) and Hoechst nuclei staining. The rows below show isolated nuclei and nuclear beta-catenin levels. Last row corresponds to Fig. 1D of the main manuscript. Scale bar 10*μ*m.
(TIF)

S3 Fig Beta-catenin levels in raft deficient cells. Microscopy images depicting beta-catenin staining (red) during early differentiation (0–12 hours) in raft-deficient ReNcell VM197 cells, treated with 2mM MbCD. The first row shows the entire cells with beta-catenin (red) and Hoechst nuclei staining. The rows below show isolated nuclei and nuclear beta-catenin levels. Last row corresponds to Fig. 1D of the main manuscript. Scale bar 10*μ*m.
(TIF)

S4 Fig Simulation result for Raft/Receptor dynamics. Representative simulation trajectory demonstrating the separation of membrane bound CK1*γ* and LRP6 molecules into lipid rafts and non-raft regions depending on their individual raft affinity. In equilibrium ∼ 85% of CK1*γ* molecules are located within rafts (LR[CK1*γ*]), whereas only ∼ 25% LRP6 molecules are raft-associated (LR[LRP6]), which corresponds to experimentally derived values in [15].
(TIF)

S1 Table Sensitivity Analysis. PRCC values for input parameters significantly correlated with model outcome (nuclear *β*-catenin concentration).
(PDF)

S1 Text Specification of ML-Rules Model and Simulation Experiments. (PDF)

S2 Text Sensitivity Analysis. (PDF)

S3 Text WNT/beta-catenin model. Source file for WNT/beta-catenin model implemented in ML-Rules.
(PDF)

S4 Text ROS/WNT/beta-catenin model. Source file for combined ROS/WNT/beta-catenin model implemented in ML-Rules.
(PDF)

S1 Datasheet Fig. 1. (CSV)

S2 Datasheet Fig. 3. (CSV)

S3 Datasheet Fig. 4. (CSV)

S4 Datasheet Fig. 6. (CSV)
