# Supplementary figures and images for: Spatio-temporal Model of Endogenous ROS and Raft-Dependent WNT/Beta-Catenin Signaling Driving Cell Fate Commitment in Human Neural Progenitor Cells
Source: PLoS Comput Biol. 2015 Mar 20;11(3):e1004106. doi: 10.1371/journal.pcbi.1004106 (PMC4368204; doi:10.1371/journal.pcbi.1004106)

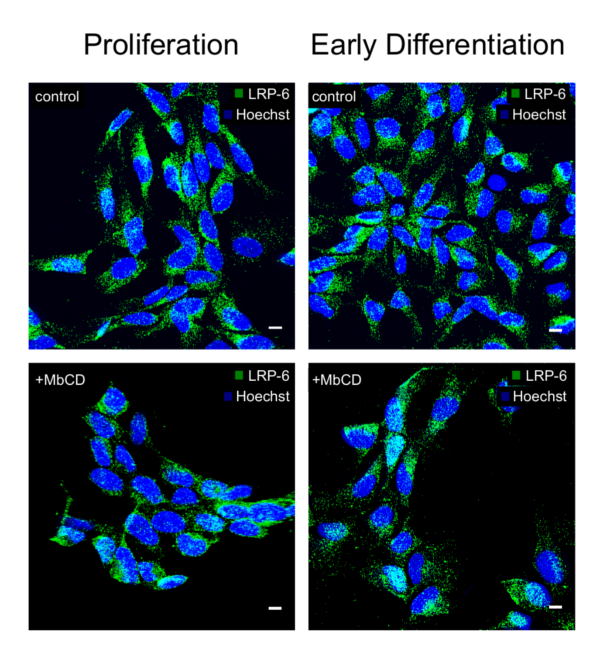

Supplement: S1 Fig — Confocal microscopy images of LRP6 staining (no Lipid Rafts staining) in proliferating and early differentiating cells. The first row shows untreated (control) cells, while cells depicted in the lower row are treated with 2mM MbCD. Scale bar 10μm (TIF) [file pcbi.1004106.s001.tif]

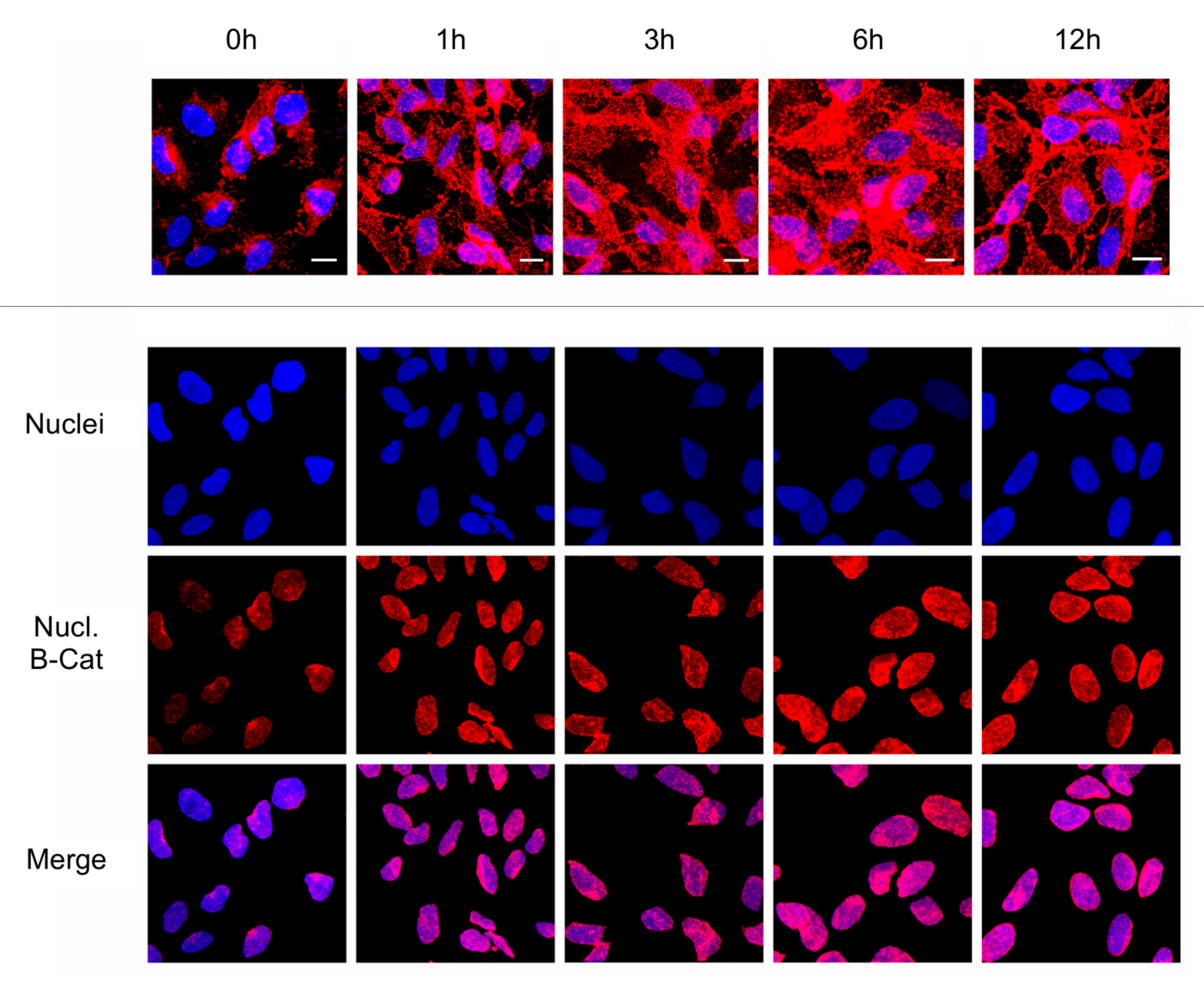

Supplement: S2 Fig — Microscopy images depicting beta-catenin staining (red) during early differentiation (0–12 hours) in non-treated ReNcell VM197 control cells. The first row shows the entire cells with beta-catenin (red) and Hoechst nuclei staining. The rows below show isolated nuclei and nuclear beta-catenin levels. Last row corresponds to Fig. 1D of the main manuscript. Scale bar 10μm. (TIF) [file pcbi.1004106.s002.tif]

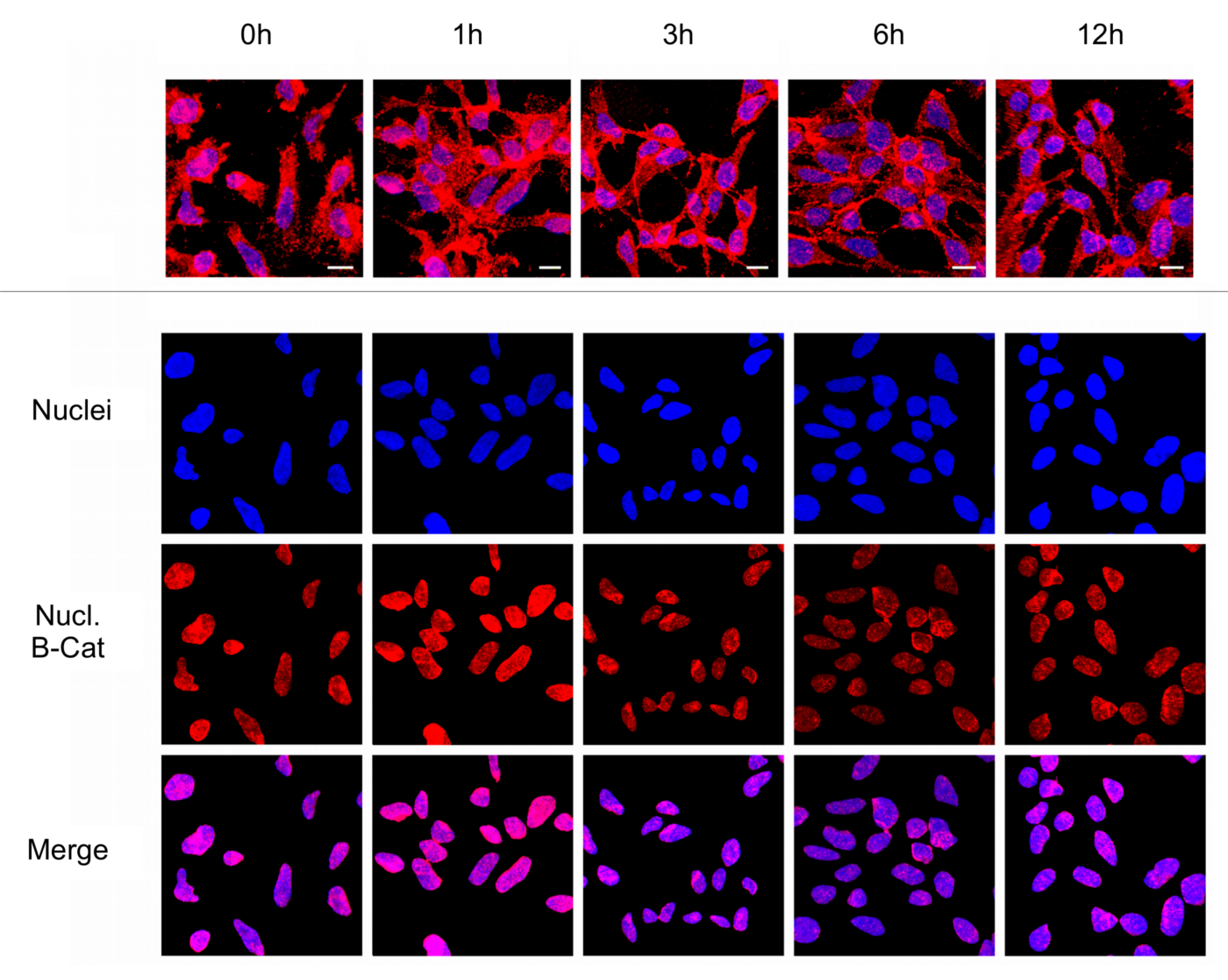

Supplement: S3 Fig — Microscopy images depicting beta-catenin staining (red) during early differentiation (0–12 hours) in raft-deficient ReNcell VM197 cells, treated with 2mM MbCD. The first row shows the entire cells with beta-catenin (red) and Hoechst nuclei staining. The rows below show isolated nuclei and nuclear beta-catenin levels. Last row corresponds to Fig. 1D of the main manuscript. Scale bar 10μm. (TIF) [file pcbi.1004106.s003.tif]

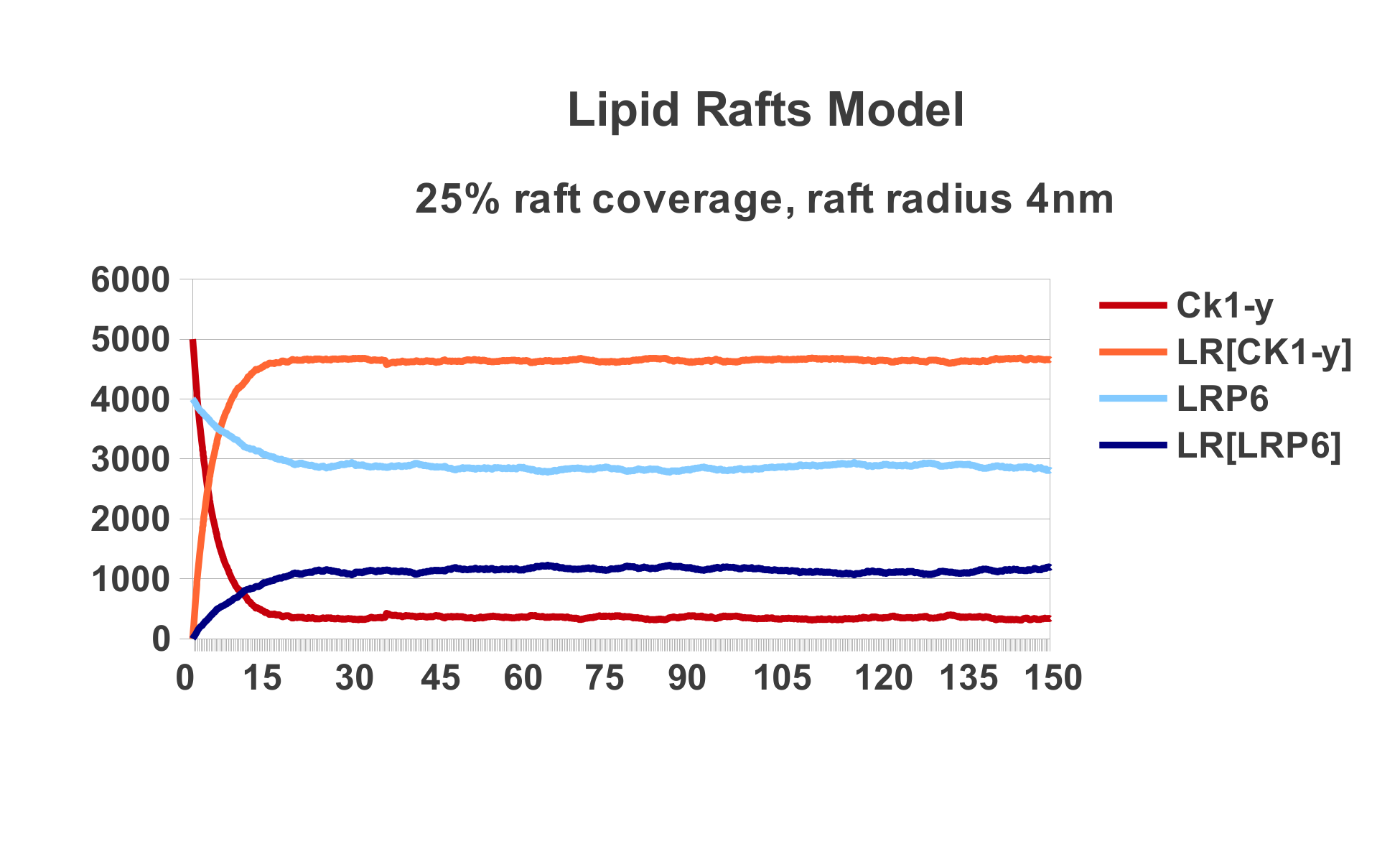

Supplement: S4 Fig — Representative simulation trajectory demonstrating the separation of membrane bound CK1γ and LRP6 molecules into lipid rafts and non-raft regions depending on their individual raft affinity. In equilibrium ∼ 85% of CK1γ molecules are located within rafts (LR[CK1γ]), whereas only ∼ 25% LRP6 molecules are raft-associated (LR[LRP6]), which corresponds to experimentally derived values in [15]. (TIF) [file pcbi.1004106.s004.tif]
