# Supplementary material for: Spatio-temporal Model of Endogenous ROS and Raft-Dependent WNT/Beta-Catenin Signaling Driving Cell Fate Commitment in Human Neural Progenitor Cells
Source: PLoS Comput Biol. 2015 Mar 20;11(3):e1004106. doi: 10.1371/journal.pcbi.1004106 (PMC4368204; doi:10.1371/journal.pcbi.1004106)
Supplement: S1 Table — PRCC values for input parameters significantly correlated with model outcome (nuclear β-catenin concentration). (PDF) [file pcbi.1004106.s005.pdf]

Table S1. Parameters significantly correlated with model outcome

1h		3h		6h		12h
kDvlAgg	0.989	kNrxNo	-0.952	kDvlAgg	-0.950	**-**
kNrxNo	-0.903	kDvlAgg	0.941	kNrxNo	0.856	
		kDvlAxinUnbind	-0.614	kDvlAxinUnbind	-0.774	
