# Supplementary material for: Spatio-temporal Model of Endogenous ROS and Raft-Dependent WNT/Beta-Catenin Signaling Driving Cell Fate Commitment in Human Neural Progenitor Cells
Source: PLoS Comput Biol. 2015 Mar 20;11(3):e1004106. doi: 10.1371/journal.pcbi.1004106 (PMC4368204; doi:10.1371/journal.pcbi.1004106)
Supplement: S1 Text — (PDF) [file pcbi.1004106.s006.pdf]

Specification of ML-Rules Model and Simulation Experiments

Model implementation in ML-Rules

In the following we give a short introduction to ML-Rules with respect to the implemented Wnt model. A model description in ML-Rules typically consists of four different elements that are specified in a certain order. We will introduce each of these elements following the ML-Rules model specification (Supplemental File 1) of the core model (cf. Figure 2) as example.

Parameter specification At first a list of optional constant model parameters is defined, like initial molecule number or reaction rate constants. In our model specification (SI-1) the initial molecule numbers are defined in lines 20–33, whereas reaction rate constants are specified in lines 39–73. Parameter names and values correspond to those listed in Table 1.

Species definition Any entity in the model is defined as species type with a unique name and its number of attributes (lines 88–106). For example $LRP6(4)$ states, that LRP6 is attributed with four different attributes: diffusion rate, raft affinity, phosphorylation state and binding state (cf. line 102). Note, that the species type definition does not contain any information about the type or the value of the attributes. Attributes are explicitly specified when defining the initial solution or the rule schemata.

Initial solution The initial model state is defined by the initial solution `>>INIT[...]`. Here, the species that are initially present, their species count and attributes are (explicitly) specified (line 112–126). Distinct species are separated by a + symbol. Note that the same species type may occur several times in the initial solution, if differently attributed. For example, in our model Axin is initially present in the phosphorylated and unphosphorylated state. Accordingly the initial solution contains two species of type Axin - one with attribute `'u'`(unphosphorylated) and one with `'p'`(phosphorylated) with the corresponding molecule counts `nAxin` and `nAxinP` (line 122–123).
Since our model is hierarchical, the initial solution of our model also comprises sub-solutions contained by certain species. The hierarchical structure is indicated by square brackets, i.e. a nested species `Cell` that encloses a `Nucleus` and a `Membrane` is, in the simplest case (without further attributes or nested structures), defined as follows:

```
Cell[Nucleus+Membrane]
```

Accordingly, in our model the initial solution comprises extracellular Wnt molecules (l. 113) and a single Cell, which holds the nested species membrane (l. 116–120) and nucleus (l. 124) and also the three molecule species β-catenin (l. 124), unphosphorylated and phoshporylation Axin (l. 122–123). While Axin is restricted to the cellular level, β-catenin is also present in the nucleus (l. 124). In addition the membrane holds the sub-solution consisting of Lipid Rafts (l. 117), LRP6 (l. 118) and CK1γ (l. 119). Note, that attributes are now explicitly specified. For example, we initialize all LRP6 molecules with diffusion speed 1, a raft affinity value (φ) of 0.15 (cf. model description), no phoshporylation and no Wnt binding. These values are changed during the simulation according to the rules defined later on.

Rule schemata Eventually the dynamics of the model are defined in terms of rules or rule schemata. A rule (schemata) consists of an arbitrary number of reactants, products and a stochastic rate. Reactants and products are separated by an arrow $\rightarrow$ followed by the rate definition after the `@` symbol:

```
reactants -> products  @ rate;
```

In our model we only consider biochemical reactions following mass action kinetics. The stochastic rate of the reaction is thus determined by the amount of reactant species and the speed of the reaction, i.e. the reaction rate constant. To access the amount of a certain reactant, it can be assigned to an identifier `x`. The corresponding special variable `#x` then holds the current number of the assigned species. For example, the simple decay reaction of Axin in our model with reaction rate constant `k11/kAdeg` is described by the following rule (l. 196):

```
Axin(phos):a -> @ kAdeg*#a;
```

where `#a` relates to the current amount of Axin molecules. At the same time this example illustrates the use of schematic rules. Instead of specifying each potential (phosphorylation) state of Axin (`'u'` and `'p'`) explicitly for its degradation, one can specify a schematic rule with a variable (`phos`) rather than a defined attribute value. This however also implies, that the degradation rate constant is the same for phosphorylated and unphosphorylated Axin. Applied to a model that comprises both species `Axin('u')` and `Axin('p')` would then lead to two rule instantiations that are equivalent to the explicitly defined rules

```
Axin('u'):a -> @ kAdeg*#a;
Axin('p'):a -> @ kAdeg*#a;
```

As indicated before, attribute values can easily be changed by putting a species to the right-hand side of the rule. Accordingly, the autophosphorylation of Axin is described with the following rule (cf. l. 193):

```
Axin('u'):a -> Axin('p') @ kAAp*#a;
```

with `kAAp` being the reaction rate constant for this reaction.
Similar to attribute values rules can be defined that describe the migration of species between various nested structures, i.e. change the location or hierarchy of a species. For example the shuttling of β-catenin between nucleus and cytosol is described in terms of the following pair of rules (l. 208 and 211):

```
Bcat:b + Nuc(vol)[s?] -> Nuc(vol)[Bcat + s?] @ kbetain*#b;
Nuc(vol)[Bcat:b + s?] -> Bcat + Nuc(vol)[s?] @ kbetaout*#b;
```

Please note, that when applying rules to nested species, one typically wants to preserve the remaining molecules (sub-solution) within the nested species (e.g. cell, nucleus or membrane) without specifying them explicitly. Therefore an additional, arbitrary variable with the suffix `?` occurs on the reactant and product site. In the example above, `s?` thus refers to the -unknown- sub-solution of the nucleus, which remains unchanged, except for the removal/addition of one β-catenin molecule. The same way, the diffusion of LRP6 and CK1γ into and out of lipid rafts is specified (l. 134–152).
Further, reactions can be restricted to a certain hierarchical level by simply stating the nested species on the reactant and product site. For example, the phosphorylation of activated LRP6, that exclusively occurs within lipid rafts, is described by the following rule:

```
Membrane(vol)[LR(radius, p)[CK1y(diff_ck, r_ck, ra_ck):ck + Lrp6(diff_l,
r_l, ra_l,    uP   ,   B   ):l + s?] + s_m?]
-> Membrane(vol)[LR(radius, p)[Lrp6(diff_l, r_l, ra_l, P,    B   ) +
CK1y(diff_ck, r_ck, ra_ck) + s?] + s_m?]
@ kLphos*#l*#ck / (3.14*radius*radius/vol) * p;
```

Specifying Simulation Experiments with SESSL

Parameter Scan A typical SESSL experiment specifying a parameter scan is shown in SESSL Code 1. After importing basic language constructs (line 1) and the support for JAMES II (l. 2), we define a file to store the results (l. 4) and execute the simulation experiment (l. 6–30). The experiment supports the observation of model variables and a parallel execution, as declared in line 7. After specifying the model file to be used (l. 8), a full factorial parameter scan is set up in lines 11–17. For each parameter either a list of values is given (e.g. `kLWntBind`), or an (inclusive) range of values is defined (e.g. `kLphos`. In line 19, JAMES II is configured to simulate the model with the ML-Rules reference implementation (see [1]). Alternatively the faster τ-leaping variant from [2] could be used as well. Then, the simulation time at which each run shall stop (l. 20), the number of replications per parameter combination (l. 21), and the number of parallel threads (l. 22) is specified. Lines 23–24 state that model variable `Cell/Nuc/Bcat()`, i.e., the number of β-catenin molecules in the nucleus, shall be observed at fixed time points, again given as a `range` of values. The last lines of the experiment specification (l. 25–28) write, for each run, the observations for `Cell/Nuc/Bcat()` into the file specified in line 4.

```
1  import sessl._
2  import sessl.james._
3
4  val modelOutput = sessl.james.tools.CSVFileWriter("./scan_Wnt_apCrine.csv")
5
6  execute {
7    new Experiment with Observation with ParallelExecution {
8      model = "file-mlrj:/." + dir + "/Wnt_apCrine.mlrj"
9
10     // Set model parameters for parameter scan:
11     scan("kLWntBind" <~ (0.01, 10, 1000))
12     scan("kLWntUnbind" <~ (0.05, 0.5, 5))
13     scan("kApA_act" <~ (0.1, 0.5, 1, 5, 10))
14     scan("kLA_diss" <~ (0.001, 0.01, 0.1, 1))
15     scan("kLWntBind" <~ (0.1, 10, 50, 100))
16     scan("kLphos" <~ range(0.1, 0.1, 1))
17     scan("kLdephos" <~ range(0.01, 0.01, 0.1))
18
19     simulator = MLRulesReference()
20     stopTime = 720
21     replications = 15
22     parallelThreads = 3
23     observe("Cell/Nuc/Bcat()") // Observe species Bcat
24     observeAt(range(1, 10, 720))
25     withRunResult { results =>
26       // Store results to file:
27       modelOutput << results.trajectory("Cell/Nuc/Bcat()")
28     }
29   }
30 }
```

SESSL Code 1

Optimization We use the Opt4J framework [3] to optimize the parameter values of our Wnt model. An exemplary optimization experiment specification is shown in SESSL Code 2. All SESSL constructs shown in SESSL Code 1. have the same meaning as explained above. The execution of the actual simulation experiment is defined in lines 11–40. It is now embedded in the call to the optimization interface of SESSL, which allows to set up a minimization experiment by defining an anonymous function that takes two arguments, `params` and `objectives` (l. 10). The `params` object (l. 10) contains the current parameters of the objective function, retrievable via `params("name")` (l. 16), and can be used to set parameters of the simulation model. The argument `objectives` represents a container to store the values of the (potentially multivariate) objective function (l. 35). Here, the objective to be minimized is the mean squared error between the simulated amount of β-catenin in the nucleus and the reference data from the wet lab (l. 8, 28–29), averaged over all replications (l. 20, 35). Note that the anonymous function to handle the results of all replications (l. 33–38) is called only once, after the last simulation replication is complete.

The second part (l. 41–53) of the optimization experiment specification determines which optimization software to use (l. 41), which parameters to optimize within which bounds (l. 42–43), and which optimization algorithm to rely on (l. 45). Optionally, Opt4J's graphical user interface allows to display intermediate results (l. 46). Again, event handlers are used to store the results of each optimization iteration (l. 47–49) and to print the overall results to standard output (l. 50–52).

References

1. Maus C, Rybacki S, Uhrmacher AM (2011) Rule-based multi-level modeling of cell biological systems. BMC Systems Biology 5: 166.

2. Helms T, Luboschik M, Schumann H, Uhrmacher AM (2013) An approximate execution of rule-based multi-level models. In: Proceedings of the 11th International Conference on Computational Methods in Systems Biology.

3. Lukasiewycz M, Glass M, Reimann F, Teich J (2011) Opt4J: a modular framework for meta-heuristic optimization. In: Proceedings of the 13th Annual Conference on Genetic and Evolutionary Computation. ACM, GECCO '11, pp. 1723–1730. doi:10.1145/2001576.2001808. URL `http://dx.doi.org/10.1145/2001576.2001808`.

```scala
import sessl._
import sessl.optimization._

import sessl.james._
import sessl.opt4j._

val optOutput = sessl.james.tools.CSVFileWriter("./opt_Wnt_aPCrine.csv")
val ref = Set(0, 7561, 8247, 7772, 7918, 7814, 7702)

minimize { (params, objective) => // Minimize the following function
  execute {
    new Experiment with Observation with ParallelExecution {
      model = "file-mlrj:/." + dir + "/Wnt_apCrine.mlrj"

      // Set model parameters as defined by optimizer:
      set("kLphos" <~ params("p"))
      set("kLdephos" <~ params("d"))

      stopTime = 721
      replications = 10
      observe("Cell/Nuc/Bcat()")
      observeAt(range(0, 120, 720))

      var runResults = 0.0 // Variables for result aggregation
      var count = 0

      withRunResult(results => {
        val numbers = results.values("Cell/Nuc/Bcat()").asInstanceOf[Iterable[Long]]
        runResults += scala.math.sqrt(mse(numbers, ref))
        count += 1
      })

      withReplicationsResult(results => {
        // Store value of objective function:
        objective <~ runResults /count
        runResults = 0.0
        count = 0
      })
    }
  }
} using (new Opt4JSetup {
  param("p", 0.1, 0.1, 10) // Optimization parameter bounds
  param("d", 0.01, 0.001, 0.1)
  // Configure optimization algorithm:
  optimizer = sessl.opt4j.SimulatedAnnealing(iterations = 15)
  // showViewer = true //Switches on Opt4J GUI
  withIterationResults { results =>
    optOutput << results
  }
  withOptimizationResults { results =>
    println("Overall results: " + results(0)) // print results to stdout
  }
})
```

SESSL Code 2
