# Supplementary material for: Spatio-temporal Model of Endogenous ROS and Raft-Dependent WNT/Beta-Catenin Signaling Driving Cell Fate Commitment in Human Neural Progenitor Cells
Source: PLoS Comput Biol. 2015 Mar 20;11(3):e1004106. doi: 10.1371/journal.pcbi.1004106 (PMC4368204; doi:10.1371/journal.pcbi.1004106)
Supplement: S2 Text — (PDF) [file pcbi.1004106.s007.pdf]

Sensitivity Analysis

To identify the parameters that significantly influence the outcome of the model and to gain information about the robustness of the newly introduced redox model with respect to our experimental results we performed a sensitivity analysis. Accordingly we used the parameter configuration in Table 2 as basis configuration and varied the parameter values of the redox-model in a range of $\pm 50\%$ while keeping parameter values of the remaining model components fixed. Due to the complex interplay between the individual parameters, it is typically not sufficient to solely vary one parameter at a time and observe the changes in the model. Instead, we apply the nearly orthogonal Latin hypercube (NOLH) approach to sample the parameter values from the given range. The NOLH is an extension of the Latin Hypercube sampling method, that provides a very good space-filling experiment design already for low numbers of parameter combinations (design points) [1, 2]. Accordingly a combination of 33 design points was sufficient to cover most relevant parts of the parameter space. As model output, we observe the nuclear beta-catenin concentrations at the same time points as our experimental measurements, i.e. at 1,3,6 and 12 hours after induction of differentiation. Thereby we can evaluate the impact of the model perturbations in a time-dependent manner allowing a more sophisticated sensitivity analysis including excitation and relaxation after the initial ROS stimulus.

To measure the correlation between parameter values and the model outcome (time dependent nuclear β-catenin concentration) we compute partial ranked correlation coefficient (PRCC) values for each time point. The ranked correlation coefficient (like PRCC) is a robust sensitivity measure, particularly for non-linear, but monotonic relationships [3]. As for normal correlation coefficients, PRCC values vary between -1 and +1 indicating perfect negative and perfect positive correlation, respectively. To assess if a PRCC is significantly different from zero, p-values derived from Student's *t-test* were calculated according to [4].

Table S1 lists all model parameters that yielded a significant PRCC value (p-value < 0.001) for each observed time point. Apparently the three parameters *kDvlAgg*, *kNrxNo* and *kDvlAxinUnbind* significantly influence the model outcome in a time dependent manner. These parameter corresponds to the key reactions of the redox-model, i.e. the oxidation of NRX after reduction by ROS (*kNrxNo*), which allows the rebinding of Dvl, the spontaneous aggregation of Dvl *kDvlAgg* and the (un)binding rate of Dvl-Axin complex, which eventually controls the amount of free Axin. Interestingly the impact of the individual parameter is strictly time dependent, as for instance the correlation sign of *kDvlAgg* and *kNrxNo* switches after 6 hours. This behavior corresponds to the excitation and the respective relaxation phase of our model in response to the transient ROS stimulus. During excitation a higher aggregation rate of (free) Dvl results in a more effective Axin inhibition and eventually in a higher nuclear beta-catenin concentration (cf. model description Figure S6 and Fig. 5). In contrast a higher NRX oxidation rate dampens the excitation, because oxidized NRX rebinds Dvl, which decreases the number of Dvl molecules available for self-aggregation and Axin binding.

However, note that the model comprises a negative feedback after which a higher beta-catenin concentration enhances the Axin synthesis. Accordingly a strong excitation results in a strong negative feedback during the relaxation phase. Therefore we observe a sudden change in the correlation coefficient of *kDvlAgg* and *kNrxNo* at 6 hours of differentiation. However, not only the sign of the correlation coefficient, but also the number of significant parameters changes between the individual time points. This is particularly apparent at 12 hours, where not a single parameter of the redox-model is significantly correlated with the model output, i.e. the nuclear beta-catenin concentration. Apparently the initial, transient ROS stimulus has been entirely processed and the model has returned to its equilibrium state, which is a first indicator for the robustness of our model with respect to ROS perturbations (cf. model configuration in Table 2).

The robustness of the model is further confirmed by the fact that ~ 30 % of all parameter configurations, match the experimental measurements of all time points.

References

1. Cioppa TM, Lucas TW (2007) Efficient nearly orthogonal and space-filling latin hypercubes. Technometrics 49: 45–55.

2. Sanchez S (2008) Better than a petaflop: The power of efficient experimental design. In: Simulation Conference, 2008. WSC 2008. Winter. pp. 73-84. doi:10.1109/WSC.2008.4736057.

3. Marino S, Hogue IB, Ray CJ, Kirschner DE (2008) A methodology for performing global uncertainty and sensitivity analysis in systems biology. Journal of Theoretical Biology 254: 178–196.

4. Anderson T (2003) An introduction to multivariate statistical analysis. Wiley series in probability and statistics. Wiley-Interscience, 3rd edition.
