# Supplementary material for: Spatio-temporal Model of Endogenous ROS and Raft-Dependent WNT/Beta-Catenin Signaling Driving Cell Fate Commitment in Human Neural Progenitor Cells
Source: PLoS Comput Biol. 2015 Mar 20;11(3):e1004106. doi: 10.1371/journal.pcbi.1004106 (PMC4368204; doi:10.1371/journal.pcbi.1004106)
Supplement: S3 Text — Source file for WNT/beta-catenin model implemented in ML-Rules. (PDF) [file pcbi.1004106.s008.pdf]

Listing 1: Source File for Wnt/beta-catenin model implemented in ML-Rules

```
/*
single-cell model (number of cells can be easily increased via parameter
    nCells),
no Wnt diffusion in space,
no cell cycle dynamics included,
compartment volumes are fixed,
*/

/*
compartment volumes:

membrane: 0.137E-16 m3
cytoplams: 8.73E-16 m3
nucleus:  3.55E-16 m3
*/

// ++++++++++++++++++++++++++++++++++
// ++++++ initial species counts ++++++
// ++++++++++++++++++++++++++++++++++

// ** Membrane signalling **
nWnt:    220;
nLRP6:   4000;
nCK1y:   5000;
nP:              1;

// ** beta-catenin signalling **
nbetacyt: 12989;
nbetanuc: 5282;
nAxin:   252;
nAxinP:  219;

nCells:  1;
nLR:     5;

// ++++++++++++++++++++++++++++++++++++++
// +++++ reaction rate coefficients +++++
// ++++++++++++++++++++++++++++++++++++++

// ** Membrane Signalling **

// Wnt
kWdeg:         0.27;
kWsyn:                 1.9;
kWdelay:       90;
kPsyn:                 1;

// LRP6
kLWntBind:         100;
kLWntUnbind:   0.1;
kLphos:                6.73E-1;
kLdephos:              4.7E-2;
kLA_diss:              3E-4;
```

```
53
54  // Lipid Rafts
55  kRin:                 1;
56  kRout:                1;
57
58  // ** beta-catenin signalling **
59
60  // Axin
61  kApA_act:     5;
62  kApA:         0.03;
63  kAAp:         0.03;
64  kAdeg:        4.48E-3;
65
66  kAsyn:        4E-4;
67
68  //beta catenin
69  kbetasyn:     600;
70  kbetadeg_act:         2.1E-4;
71  kbetadeg:     1.13E-4;
72  kbetain:      0.0549;
73  kbetaout:     0.135;
74
75  // diffusion coefficient
76  D:1;
77
78  // raft fluidity
79  rho:0.1;
80
81  // raft radius
82  radius:4;
83
84  // threshold wnt production, corresponds to rounded values of those
        listed in Figure 3C of main manuscript, e.g. 10.35 -> 11.
85  // used only in validation experiments
86  // epsilonW: 11;
87
88  // ++++++++++++++++++++++++++++++++++++++++++++++++++++++++
89  // +++++ species definitions (number of attributes) +++++
90  // ++++++++++++++++++++++++++++++++++++++++++++++++++++++++
91
92  // legend:
93  // [species name](number of attributes); // attr1 (variable - values -
        comment) | attr2 (variable - values - comment) ....
94
95
96  Cell(2); // cell cycle phase (phase - 'G1' - dummy for dynamic cell
        cycle states) | cytosolic compartment volume (vol - 1 - dummy for
        dynamic compartment volumes, e.g. growth processes)
97  Membrane(1); // area (A - 1000 - arbitrary unit, required for dynamic
        rate calculation wrt. raft-related processes)
98  Nuc(1); // volume (vol - 1 - dummy for dynamic compartment volumes, e.g.
        growth processes)
99  Wnt(0);
100 Bcat(0);
```

```
101 Axin(1); // phosphorylation state (x - 'u' & 'p' - phosphorylation state
        of Axin)
102 Lrp6(4); // diffusion rate (d - 1 & 0.1 - diffusion speed of LRP6,
        depending on localization) | raft affinity (ra - 0.15 - raft
        affinity of LRP6) | phosphorylation state (phos - 'uP' & 'P' -
        phosphorylation state of LRP6) | binding state (bind - 'uB' & 'B' -
        binding state of LRP6-Wnt complex)
103 Lrp6Axin(3); // phosphorylation state (phos - 'uP' & 'P' -
        phosphorylation state of axin) | diffusion rate (d - 1 & 0.1 -
        diffusion speed of LRP6-Axin comlex depending on localization) |
        raft affinity (ra - 0.15 - raft affinity of LRP6)
104 CK1y(2); // diffusion rate (d - 1 & 0.1 - diffusion speed of CK1y
        depending on localization) | raft affinity (ra - 1 - raft affinity
        of CK1y)
105 P(0);
106 LR(2); // radius (radius - 4 - arbitrary unit, required for dynamic
        rate calculation) | fluidity (rho - 0.1 - raft fluidity determines
        the slow-down of raft-associated receptors)
107
108
109 // ++++++++++++++++++++++++++++
110 // +++++ initial solution +++++
111 // ++++++++++++++++++++++++++++
112 >>INIT[
113        (nWnt) Wnt +
114        (nP) P +
115    nCells Cell('G1', 1)[
116          (1) Membrane(1000)[
117            nLR LR(radius, rho) +
118                nLRP6 Lrp6(1, 0.15, 'uP', 'uB') +
119                nCK1y CK1y(1, 1)
120          ] +
121        (nbetacyt) Bcat +
122        nAxin Axin('u') +
123        nAxinP Axin('p') +
124        Nuc(1)[(nbetanuc) Bcat]
125        ]
126 ];
127
128 // ++++++++++++++++++++++++++++++
129 // +++++ reaction rules ++++++++++
130 // ++++++++++++++++++++++++++++++
131
132 // ********** Lipid Raft Dynamics ***************
133
134 // (R1) Lrp6 diffusion into lipid rafts
135 // note the change of diffusion rate of LRP6 due to raft entry
136 Membrane(A)[LR(radius, p)[s?]:l + Lrp6(d, ra, phos, bind):r + s_m?] ->
        Membrane(A)[LR(radius, p)[Lrp6(d*p, ra, phos, bind) + s?] + s_m?] @
    ra*kRin*(4*3.14*d*radius*#l)*(#r/(v-(3.14*radius*radius)));
137
138
139 // (R2)Lrp6 diffusion out of lipid rafts
140 // note the change of diffusion rate LRP6 due to raft exit
141 Membrane(A)[LR(radius, p)[Lrp6(d, ra, phos, bind):r + s?]:l + s_m?] ->
```

```
    Membrane(A)[LR(radius, p)[s?] + Lrp6(d/p, ra, phos, bind) + s_m?] @
142 kRout*(4*3.14*d*radius*#l)*(#r/(3.14*radius*radius));
143
144 // (R3) CK1y diffusion into lipid rafts
145 // note the change of diffusion rate CK1y due to raft entry
146 Membrane(A)[LR(radius, p)[s?]:l + CK1y(d, ra):r + s_m?] ->
    Membrane(A)[LR(radius, p)[CK1y(d*p, ra) + s?] + s_m?] @
147 ra*kRin*(4*3.14*d*radius*#l)*(#r/(v-(3.14*radius*radius)));
148
149 // (R4) CK1y diffusion out of lipid rafts
150 // note the change of diffusion rate CK1y due to raft exit
151 LR(radius, p)[CK1y(d, ra):r + s?]:l -> LR(radius, p)[s?] + CK1y(d/p, ra)
    @
152 kRout*(4*3.14*d*radius*#l)*(#r/(3.14*radius*radius));
153
154 // **** Membrane Signalling ****
155
156
157 // (R5) Pseudoparticle production
158 // Note, that it is (not yet) possible to specify delays explicitly.
159 // Therefore we have to find a workaround to schedule the production of
    Wnt after a certain amount of time
160 // This is done by counting a "pseudoparticle", of which exactly one
    particle is synthezised at every time step (see next rule)
161 Cell(phase,vol)[s?] -> P + Cell(phase,vol)[s?] @ kPsyn;
162
163 // (R6) Wnt production
164 P:p + Cell(phase,vol)[s?] -> Wnt + P + Cell(phase,vol)[s?] @ if
    ((#p>kWdelay)) then kWsyn else 0;
165
166 // (R6a) Wnt production, restricted to certain threshold concentration -
    used for validation experiment
167 // P:p + Cell(phase,vol)[s?] + Wnt:w -> Wnt + P + Cell(phase,vol)[s?] @
    if ((#w<epsilon)) then kWsyn else 0;
168
169 // (R7) Wnt degradation
170 Wnt:w -> @ kWdeg*#w;
171
172 // (R8) Binding of Wnt to Lrp6 (representing Fz,Lrp6 receptor complex)
173 Wnt:w + Cell(S, vol)[Membrane(A)[Lrp6(diff, ra, 'uP', 'uB'):l + sm?] +
    s?] -> Cell(S, vol)[Membrane(A)[Lrp6(diff, ra, 'uP', 'B') + sm?] +
    s?]
174  @ kLWntBind*#w*#l;
175
176 // (R9) Dissociation of Wnt from LRP6 (representing Fz, Lrp6 receptor
    complex)
177 Cell(S, vol)[Membrane(A)[Lrp6(diff, ra, 'uP', 'B'):l + sm?] + s?] ->
    Cell(S, vol)[Membrane(A)[Lrp6(diff, ra, 'uP', 'uB') + sm?] + s?] +
    Wnt @ kLWntUnbind*#l;
178
179 // (R10) Phosphorylation of activated Lrp6 in LR
180 Membrane(vol)[LR(radius, p)[CK1y(diff_ck, ra_ck):ck + Lrp6(diff_l, ra_l,
    'uP','B'):l + s?] + s_m?]
181 -> Membrane(vol)[LR(radius, p)[Lrp6(diff_l, ra_l, 'P', 'B') +
```

```
    CK1y(diff_ck, ra_ck) + s?] + s_m?]
@ kLphos*#l*#ck / (3.14*radius*radius/vol) * p;

// (R11) Dephosphorylation of Lrp6
Lrp6(diff, ra, 'P', 'B'):l -> Lrp6(diff, ra, 'uP', 'B') @ kLdephos*#l;

// **** Beta-catenin signalling ****

// (R12) Basal AxinP dephosphorylation
Axin('p'):a -> Axin('u') @ kApA*#a;

// (R13) Axin phosphorylation
Axin('u'):a -> Axin('p') @ kAAp*#a;

// (R14) Axin degradation
Axin(phos):a -> @ kAdeg*#a;

// (R15) Activated beta-catenin degradation
Cell(phase,vol)[Axin('p'):a + Bcat:b + s?]:c ->
    Cell(phase,vol)[Axin('p') + s?] @ #c*((kbetadeg_act*#a*#b));

// (R16) Beta-catenin synthesis
Cell(phase,vol)[s?]:c -> Cell(phase,vol)[Bcat + s?] @ #c*kbetasyn;

// (R17) Basal beta-catenin degradation
Bcat:b -> @ kbetadeg*#b;

// (R18) Beta-catenin shuttling into the nucleus
Bcat:b + Nuc(vol)[s?] -> Nuc(vol)[Bcat + s?] @ kbetain*#b;

// (R19) Beta-catenin shuttling out of the nucleus
Nuc(vol)[Bcat:b + s?] -> Bcat + Nuc(vol)[s?] @ kbetaout*#b;

// (R20) Axin synthesis
Nuc(vol)[Bcat:b + s?] -> Nuc(vol)[Bcat + s?] + Axin('u') @ kAsyn*#b;

// **** Axin LRP6 signalling ****

// (R21) Axin binding by LRP6 in membrane
Axin(phos):a + Membrane(vol)[Lrp6PP(diff, ra, 'P','B'):l + s?] ->
    Membrane(vol)[Lrp6Axin(phos, diff, ra) + s?] @ ((kApA_act*#l*#a));

// (R22) Axin binding by LRP6 in lipid rafts
Axin(phos):a + Membrane(vol)[LR(radius_lr, p)[Lrp6PP(diff, ra,
    'P','B'):l + s_lr?] + s?] ->
Membrane(vol)[LR(radius_lr, p)[Lrp6Axin(phos, diff, ra) + s_lr?] + s?] @
    ((kApA_act*#l*#a));

// (R23) Dissociation of receptor/Axin complex (signalosome) in membrane
Cell(phase, vol)[Membrane(vol_m)[Lrp6Axin(phos, diff, ra):la + s_m?] +
    s?] ->
Cell(phase, vol)[Membrane(vol_m)[Lrp6(diff, ra, 'uP', 'uB') + s_m?] +
    Axin(phos) + s?] @(kLA_diss)*#la;

```

```
// (R24) Dissociation of receptor/Axin complex (signalosome) in LR
Cell(phase, vol)[Membrane(vol_m)[LR(radius_lr, p)[Lrp6Axin(phos, diff,
    ra):la + s_lr?] + s_m?] + s?] ->
Cell(phase, vol)[Membrane(vol_m)[LR(radius_lr, p)[Lrp6(diff, ra, 'uP',
    'uB') + s_lr?] + s_m?] + Axin(phos) + s?] @(kLA_diss)*#la;
```
