# Supplementary material for: Spatio-temporal Model of Endogenous ROS and Raft-Dependent WNT/Beta-Catenin Signaling Driving Cell Fate Commitment in Human Neural Progenitor Cells
Source: PLoS Comput Biol. 2015 Mar 20;11(3):e1004106. doi: 10.1371/journal.pcbi.1004106 (PMC4368204; doi:10.1371/journal.pcbi.1004106)
Supplement: S4 Text — Source file for combined ROS/WNT/beta-catenin model implemented in ML-Rules. (PDF) [file pcbi.1004106.s009.pdf]

Listing 1: Source File for entire Ros/Wnt/beta-catenin model implemented in ML-Rules

```
/*
single-cell model (number of cells can be easily increased via parameter nCells),
no Wnt diffusion in space,
no cell cycle dynamics included,
compartment volumes are fixed,
*/

/*
compartment volumes:

membrane: 0.137E-16 m3
cytoplams: 8.73E-16 m3
nucleus:  3.55E-16 m3
*/

// ++++++++++++++++++++++++++++++++++++
// ++++++ initial species counts ++++++
// ++++++++++++++++++++++++++++++++++++

// ** Membrane signalling **
nWnt:   0;
nLRP6:    4000;
nCK1y:   5000;
nP:             1;

// ** Ros-Dvl signalling **
nRos: 10000;
nDvl: 855;
nNrx: 18;
nDvlNrx: 36200;

// ** beta-catenin signalling **
nbetacyt: 12989;
nbetanuc: 5282;
nAxin:   252;
nAxinP:  219;

nCells:  1;
nLR:     5;

// ++++++++++++++++++++++++++++++++++++++
// +++++ reaction rate coefficients +++++
// ++++++++++++++++++++++++++++++++++++++

// ** Membrane Signalling **

// Wnt
kWdeg:        0.27;
kWsyn:               1.9;
kWdelay:             90;
kPsyn:               1;
```

```
// LRP6
kLWntBind:        100;
kLWntUnbind:   0.1;
kLphos:               6.73E-1;
kLdephos:             4.7E-2;
kLA_diss:             3E-4;

kLd: 3.5E4;
kLad: 3.5E-4;

// Lipid Rafts
kRin:                 1;
kRout:                1;

// ** Ros-Dvl Signalling **
kRosSyn:              0;
kRosDelay: 0;
kRosAct:              7.2E2;

// Nrx
kNrxRos:       5E2;
kNrxNo:        2E-2;

// Dvl
kDvlSponAgg: 5E-04;
kDvldisAgg: 0.5;

// Dvl-Nrx
kDvlNrxBind:   22.5;
kDvlNrxUnbind: 2.3E-2;
kDvlNrxRos:    3.2E2;

// ** beta-catenin signalling **

// Axin
kApA_act:      5;
kApA:          0.03;
kAAp:          0.03;
kAdeg:         4.48E-3;

kAsyn:         4E-4;

kDvlAxinBind:  0.075;
kDvlAxinUnbind: 6.8E-2;

//beta catenin
kbetasyn:      600;
kbetadeg_act:         2.1E-4;
kbetadeg:      1.13E-4;
kbetain:       0.0549;
kbetaout:      0.135;

// diffusion coefficient
```

```
D:1;

// raft fluidity
rho:0.1;

//raft radius
radius: 4;

// threshold wnt production, corresponds to rounded values of those listed in Figure 3C of main manuscript, e.g. 10.35 -> 11.
// used only in validation experiments
// epsilonW: 11;


// ++++++++++++++++++++++++++++++++++++++++++++++++++++++++
// +++++ species definitions (number of attributes) +++++
// ++++++++++++++++++++++++++++++++++++++++++++++++++++++++

// legend:
// [species name](number of attributes); // attr1 (variable - values - comment) | attr2 (variable - values - comment) ....


Cell(2); // cell cycle phase (phase - 'G1' - dummy for dynamic cell cycle states) | cytosolic compartment volume (vol - 1 - dummy for dynamic compartment volumes, e.g. growth processes)
Membrane(1); // area (A - 1000 - arbitrary unit, required for dynamic rate calculation wrt. raft-related processes)
Nuc(1); // volume (vol - 1 - dummy for dynamic compartment volumes, e.g. growth processes)
Wnt(0);
Bcat(0);
Axin(1); // phosphorylation state (x - 'u' & 'p' - phosphorylation state of Axin)
Lrp6(4); // diffusion rate (d - 1 & 0.1 - diffusion speed of LRP6, depending on localization) | raft affinity (ra - 0.15 - raft affinity of LRP6) | phosphorylation state (phos - 'uP' & 'P' - phosphorylation state of LRP6) | binding state (bind - 'uB' & 'B' - binding state of LRP6-Wnt complex)
Lrp6Dvl(4); // phosphorylation state (phos - 'uP' & 'P' - phosphorylation state of LRP6) | diffusion rate (d - 1 & 0.1 - diffusion speed of LRP6-Dvl comlex depending on localization) | raft affinity (ra - 0.15 - raft affinity of LRP6) | binding state (bind - 'uB' & 'B' - binding state of LRP6-Wnt complex)
Lrp6Axin(3); // phosphorylation state (phos - 'uP' & 'P' - phosphorylation state of axin) | diffusion rate (d - 1 & 0.1 - diffusion speed of LRP6-Axin comlex depending on localization) | raft affinity (ra - 0.15 - raft affinity of LRP6)
CK1y(2); // diffusion rate (d - 1 & 0.1 - diffusion speed of CK1y depending on localization) | raft affinity (ra - 1 - raft affinity of CK1y)

Dvl(1); // aggregation state (a - 'i' & 'a' - states whether Dvl is present in aggregated or 'single' form)
```

```
Nrx(1); // oxidation state ( - 'nO' & 'O' - oxidation state based on ROS interaction)
DvlNrx(0);
DvlAxin(1); // phosphorylation state (phos - 'uP' & 'P' - phosphorylation state of axin)
Ros(1); // activation state ( - 'i' & 'a' - auxhiliary structure to control the release of ros)

P(0);
LR(2); // radius (radius - 4 - arbitrary unit, required for dynamic rate calculation) | fluidity (rho - 0.1 - raft fluidity determines the slow-down of raft-associated receptors)


// +++++++++++++++++++++++++++++
// +++++ initial solution +++++
// +++++++++++++++++++++++++++++

>>INIT[
       (nWnt) Wnt +
       (nP) P +
    nCells Cell('G1', 1)[
         (1) Membrane(1000)[
           nLR LR(radius, rho) +
               nLRP6 Lrp6(1, 'm', 0.15, 'uP', 'uB') +
               nCK1y CK1y(1, 'm', 1)
         ] +
       (nDvl) Dvl('i') +
       (nNrx) Nrx('nO') +
       (nDvlNrx) DvlNrx +
       (nRos) Ros('i') +
       (nbetacyt) Bcat +
       nAxin Axin('u') +
       nAxinP Axin('p') +
       Nuc(1)[(nbetanuc) Bcat]
       ]
];

// ++++++++++++++++++++++++++++++++
// +++++ reaction rules ++++++++++
// ++++++++++++++++++++++++++++++++

// ********** Lipid Raft Dynamics ***************

// (R1) Lrp6 diffusion into lipid rafts
// note the change of diffusion rate of LRP6 due to raft entry
Membrane(A)[LR(radius, p)[s?]:l + Lrp6(d, ra, phos, bind):r + s_m?] -> Membrane(A)[LR(radius, p)[Lrp6(d*p, ra, phos, bind) + s?] + s_m?] @
ra*kRin*(4*3.14*d*radius*#l)*(#r/(v-(3.14*radius*radius)));

// (R2) Lrp6 diffusion out of lipid rafts
// note the change of diffusion rate LRP6 due to raft exit
Membrane(A)[LR(radius, p)[Lrp6(d, ra, phos, bind):r + s?]:l + s_m?] -> Membrane(A)[LR(radius, p)[s?] + Lrp6(d/p, ra, phos, bind) + s_m?] @
```

```
kRout*(4*3.14*d*radius*#l)*(#r/(3.14*radius*radius));

// (R3) CK1y diffusion into lipid rafts
// note the change of diffusion rate CK1y due to raft entry
Membrane(A)[LR(radius, p)[s?]:l + CK1y(d, ra):r + s_m?] -> Membrane(A)[LR(radius, p)[CK1y(d*p, ra) + s?] + s_m?] @
ra*kRin*(4*3.14*d*radius*#l)*(#r/(v-(3.14*radius*radius)));

// (R4) CK1y diffusion out of lipid rafts
// note the change of diffusion rate CK1y due to raft exit
LR(radius, p)[CK1y(d, ra):r + s?]:l -> LR(radius, p)[s?] + CK1y(d/p, ra) @
kRout*(4*3.14*d*radius*#l)*(#r/(3.14*radius*radius));


// **** Membrane Signalling ****

// (R5) Pseudoparticle production
// Note, that it is (not yet) possible to specify delays explicitly.
// Therefore we have to find a workaround to schedule the production of Wnt after a certain amount of time
// This is done by counting a "pseudoparticle", of which exactly one particle is synthezised at every time step (see next rule)
Cell(phase,vol)[s?] -> P + Cell(phase,vol)[s?] @ kPsyn;

// (R6) Wnt production
P:p + Cell(phase,vol)[s?] -> Wnt + P + Cell(phase,vol)[s?] @ if ((#p>kWdelay)) then kWsyn else 0;

// (R6a) Wnt production, restricted to certain threshold concentration - used for validation experiment
// P:p + Cell(phase,vol)[s?] + Wnt:w -> Wnt + P + Cell(phase,vol)[s?] @ if ((#w<epsilon)) then kWsyn else 0;

// (R7) Wnt degradation
Wnt:w -> @ kWdeg*#w;

// (R8) Binding of Wnt to Lrp6 (representing Fz,Lrp6 receptor complex)
Wnt:w + Cell(S, vol)[Membrane(A)[Lrp6(diff, ra, 'uP', 'uB'):l + sm?] + s?] -> Cell(S, vol)[Membrane(A)[Lrp6(diff, ra, 'uP', 'B') + sm?] + s?]
 @ kLWntBind*#w*#l;

// (R9) Dissociation of Wnt from LRP6 (representing Fz, Lrp6 receptor complex)
Cell(S, vol)[Membrane(A)[Lrp6(diff, ra, 'uP', 'B'):l + sm?] + s?] -> Cell(S, vol)[Membrane(A)[Lrp6(diff, ra, 'uP', 'uB') + sm?] + s?] + Wnt @ kLWntUnbind*#l;

// (R10) Phosphorylation of activated Lrp6 in LR
Membrane(vol)[LR(radius, p)[CK1y(diff_ck, ra_ck):ck + Lrp6(diff_l, ra_l, 'uP','B'):l + s?] + s_m?]
-> Membrane(vol)[LR(radius, p)[Lrp6(diff_l, ra_l, 'P', 'B') + CK1y(diff_ck, ra_ck) + s?] + s_m?]
```

```
@ kLphos*#l*#ck / (3.14*radius*radius/vol) * p;

// (R11) Dephosphorylation of Lrp6
Lrp6(diff, ra, ’P’, ’B’):l -> Lrp6(diff, ra, ’uP’, ’B’) @ kLdephos*#l;

// **** Beta-catenin signalling ****

// (R12) Basal AxinP dephosphorylation
Axin(’p’):a -> Axin(’u’) @ kApA*#a;

// (R13) Axin phosphorylation
Axin(’u’):a -> Axin(’p’) @ kAAp*#a;

// (R14) Axin degradation
Axin(phos):a -> @ kAdeg*#a;

// (R15) Activated beta-catenin degradation
Cell(phase,vol)[Axin(’p’):a + Bcat:b + s?]:c ->
    Cell(phase,vol)[Axin(’p’) + s?] @ #c*((kbetadeg_act*#a*#b));

// (R16) Beta-catenin synthesis
Cell(phase,vol)[s?]:c -> Cell(phase,vol)[Bcat + s?] @ #c*kbetasyn;

// (R17) Basal beta-catenin degradation
Bcat:b -> @ kbetadeg*#b;

// (R18) Beta-catenin shuttling into the nucleus
Bcat:b + Nuc(vol)[s?] -> Nuc(vol)[Bcat + s?] @ kbetain*#b;

// (R19) Beta-catenin shuttling out of the nucleus
Nuc(vol)[Bcat:b + s?] -> Bcat + Nuc(vol)[s?] @ kbetaout*#b;

// (R20) Axin synthesis
Nuc(vol)[Bcat:b + s?] -> Nuc(vol)[Bcat + s?] + Axin(’u’) @ kAsyn*#b;

// **** Axin LRP6 signalling ****

// (R21) Axin binding by LRP6 in membrane
Axin(phos):a + Membrane(vol)[Lrp6PP(diff, ra, ’P’,’B’):l + s?] ->
    Membrane(vol)[Lrp6Axin(phos, diff, ra) + s?] @ ((kApA_act*#l*#a));

// (R22) Axin binding by LRP6 in lipid rafts
Axin(phos):a + Membrane(vol)[LR(radius_lr, p)[Lrp6PP(diff, ra,
    ’P’,’B’):l + s_lr?] + s?] ->
Membrane(vol)[LR(radius_lr, p)[Lrp6Axin(phos, diff, ra) + s_lr?] + s?] @
    ((kApA_act*#l*#a));

// (R23) Dissociation of receptor/Axin complex (signalosome) in membrane
Cell(phase, vol)[Membrane(vol_m)[Lrp6Axin(phos, diff, ra):la + s_m?] +
    s?] ->
Cell(phase, vol)[Membrane(vol_m)[Lrp6(diff, ra, ’uP’, ’uB’) + s_m?] +
    Axin(phos) + s?] @(kLA_diss)*#la;

// (R24) Dissociation of receptor/Axin complex (signalosome) in LR
```

```
Cell(phase, vol)[Membrane(vol_m)[LR(radius_lr, p)[Lrp6Axin(phos, diff, ra):la + s_lr?] + s_m?] + s?] ->
Cell(phase, vol)[Membrane(vol_m)[LR(radius_lr, p)[Lrp6(diff, ra, 'uP', 'uB') + s_lr?] + s_m?] + Axin(phos) + s?] @(kLA_diss)*#la;

// (R25) Binding of Dvl to LRP6/Wnt complex
Cell(S, vol)[Membrane(A)[LR(radius, p)[Lrp6(diff, r, ra, 'uP', 'B'):l + sr?] + sm?] + Dvl(a):d + s?]
-> Cell(S, vol)[Membrane(A)[LR(radius, p)[Lrp6Dvl(diff, r, ra, 'uP', 'B') + sr?] + sm?] + s?] @ kLd*#l*#d;

// (R26) Unbinding of Dvl from LRP6/Wnt complex
Cell(S, vol)[Membrane(A)[LR(radius, p)[Lrp6Dvl(diff, r, ra, 'uP', 'B'):l + sr?] + sm?] + s?] ->
Cell(S, vol)[Membrane(A)[LR(radius, p)[Lrp6(diff, r, ra, 'uP', 'B') + sr?] + sm?] + Dvl('i') + s?] @ kLad*#l;

// **** Ros-Dvl Signalling ****

// (R27) Ros Synthesis
Cell(phase, vol)[s?] -> Cell(phase, vol)[Ros('a') + s?] @ kRosSyn;

// (R28) Ros activation after delay
Cell(phase, vol)[Ros('i'):r + s?] + P:p -> Cell(phase, vol)[Ros('a') + s?] + P @ if ((#p>kRosDelay)) then kRosAct*#r else 0;

// (R29) Oxidation of Nrx by Ros
Cell(phase, vol)[Nrx('nO'):n + Ros('a'):r + s?] -> Cell(phase, vol)[Nrx('O') + s?] @ kNrxRos*#n*#r;

// (R30) Reduction of Nrx
Nrx('O'):n -> Nrx('nO') @ kNrxNo*#n;

// (R31) Activation (by e.g. aggregation) of Dvl
Dvl('i'):d -> Dvl('a') @ kDvlSponAgg*#d;

// (R32) Dynamic deactivation (e.g. by disaggregation) of Dvl
Dvl('a'):d -> Dvl('i') @ kDvldisAgg*#d;

// (R33) Forced Disaggregation of Dvl by un-oxidized Nrx
Cell(phase, vol)[Dvl('a'):d + Nrx('nO'):n + s?] -> Cell(phase, vol)[DvlNrx + s?] @kDvlNrxBind*#d*#n ;

// (R34) Binding of Dvl by Nrx
Cell(phase, vol)[Dvl('i'):d + Nrx('nO'):n + s?] -> Cell(phase, vol)[DvlNrx + s?] @ kDvlNrxBind*#d*#n;

// (R35) Basal unbinding of Dvl from Nrx
DvlNrx:dn -> Dvl('i') + Nrx('nO') @ kDvlNrxUnbind*#dn;

// (R36) Unbinding of Dvl from Nrx by Ros
Cell(phase, vol)[DvlNrx:dn + Ros('a'):r + s?] -> Cell(phase, vol)[Dvl('i') + Nrx('O') + s?] @kDvlNrxRos*#dn*#r;

```

```
// **** Axin Dvl signalling ****

// (R37) Axin binding by activated Dvl
Cell(phase, vol)[Dvl('a'):d + Axin(phos):a + s?] -> Cell(phase,
    vol)[DvlAxin(phos) + s?] @kDvlAxinBind*#d*#a;

// (R38) Axin Dvl unbinding
Cell(phase, vol)[DvlAxin(phos):da + s?] -> Cell(phase, vol)[Dvl('a') +
    Axin(phos) + s?] @kDvlAxinUnbind*#da;
```
